# Supplementary material for: Diverse protein assembly driven by metal and chelating amino acids with selectivity and tunability
Source: Nat Commun. 2019 Dec 5;10:5545. doi: 10.1038/s41467-019-13491-w (PMC6895169; doi:10.1038/s41467-019-13491-w)
Supplement: Supplementary file 1 — Supplementary Information [file 41467_2019_13491_MOESM1_ESM.pdf]

Supplementary Information for

Diverse Protein Assembly Driven by Metal and Chelating Amino Acids with Selectivity and Tunability

by Yang et al.

Table of contents

Supplementary Figures

1. Synthesis of Bpy-Ala: Supplementary Figure 1-5 3-7
2. Sample preparations: Supplementary Figure 6 8
3. 1D-assembly data: Supplementary Figure 7-35 9-37
4. 2D-assembly data: Supplementary Figure 36-43 38-45
5. Combinatory and hierarchical structures: Supplementary Figure 44-46 46-48
6. Stability and steady-state activity assays: Supplementary Figure 47-49 49-51

Supplementary Tables

1. Supplementary Table 1-3 52-54

Supplementary Notes

1. Supplementary Note 1 55

Supplementary References 56

Supplementary Figures

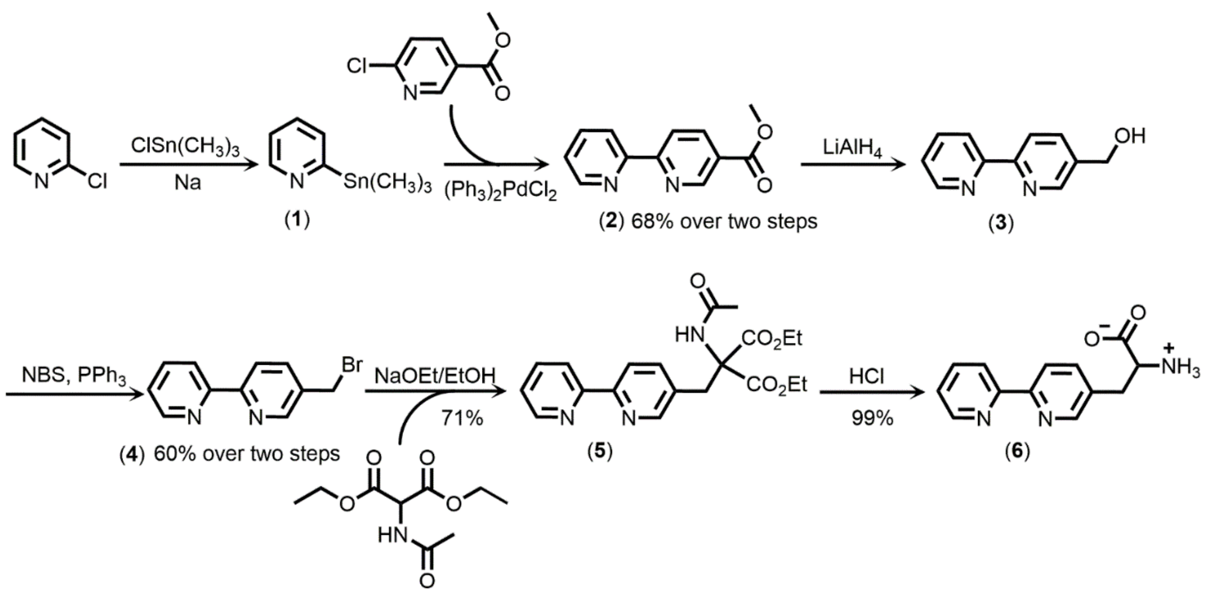

Supplementary Figure 1. Synthesis of Bpy-Ala

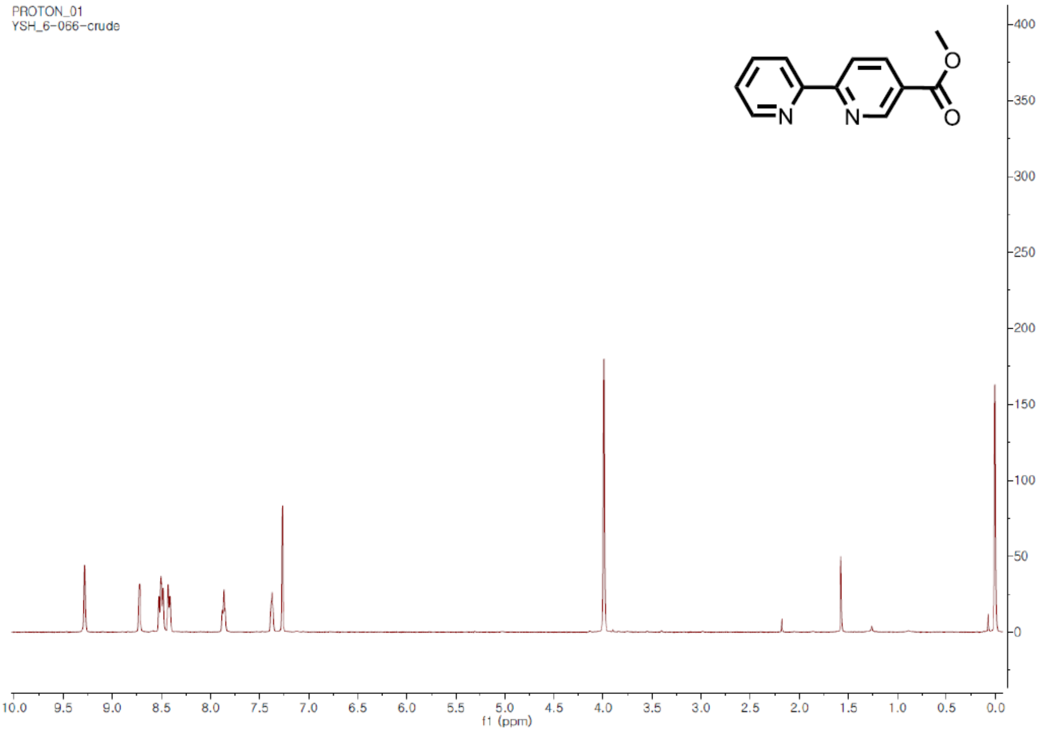

Supplementary Figure 2. NMR spectrum of 2,2´- bipyridine-5-carboxylate **(2)**

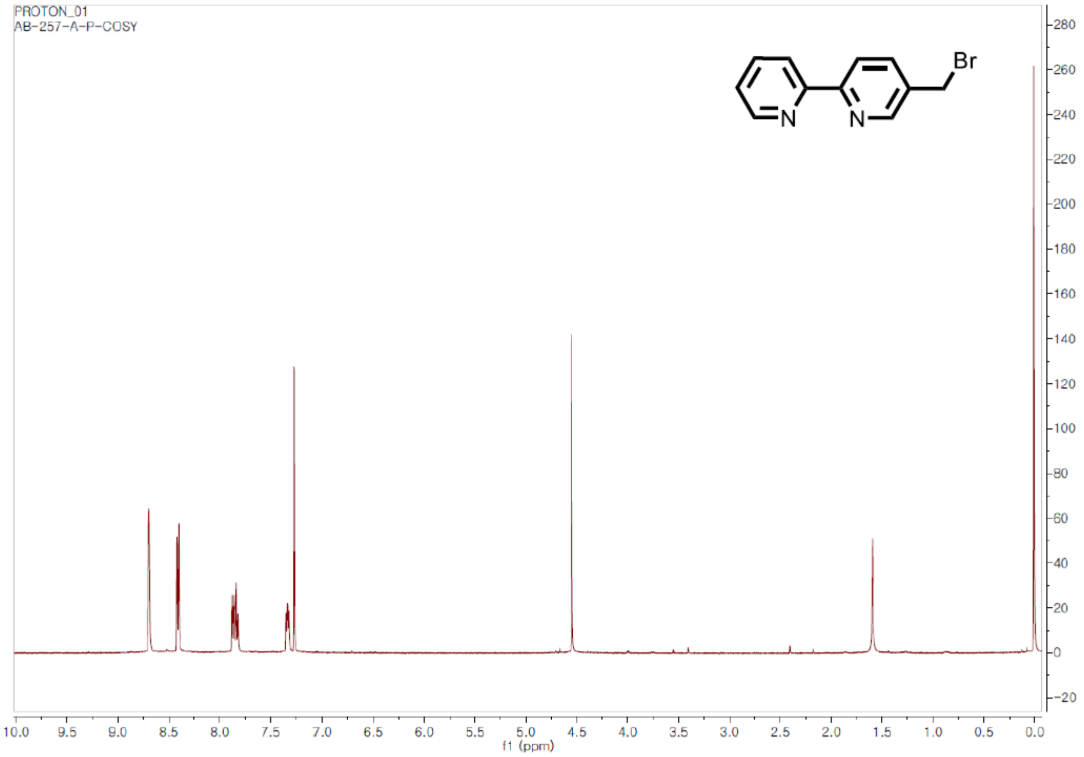

Supplementary Figure 3. NMR spectrum of 5-(bromomethyl) 2,2´- bipyridine **(4)**

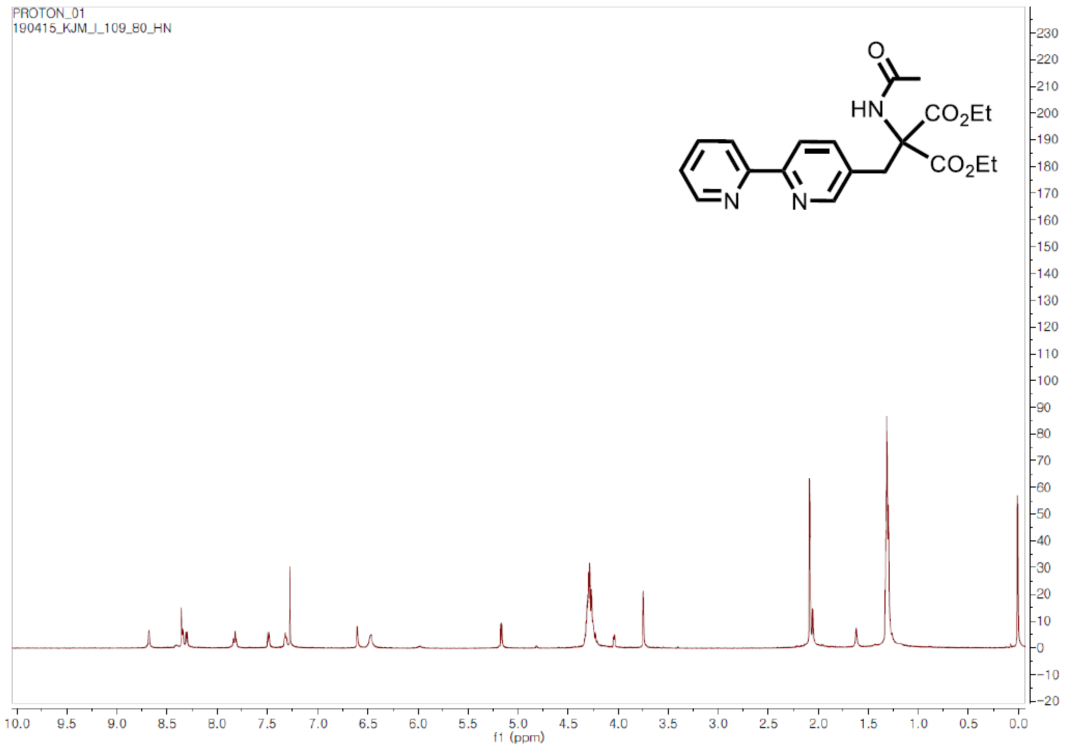

Supplementary Figure 4. NMR spectrum of (2,2′-bipyridin-5-yl)diethyl acetacetomalonate **(5)**

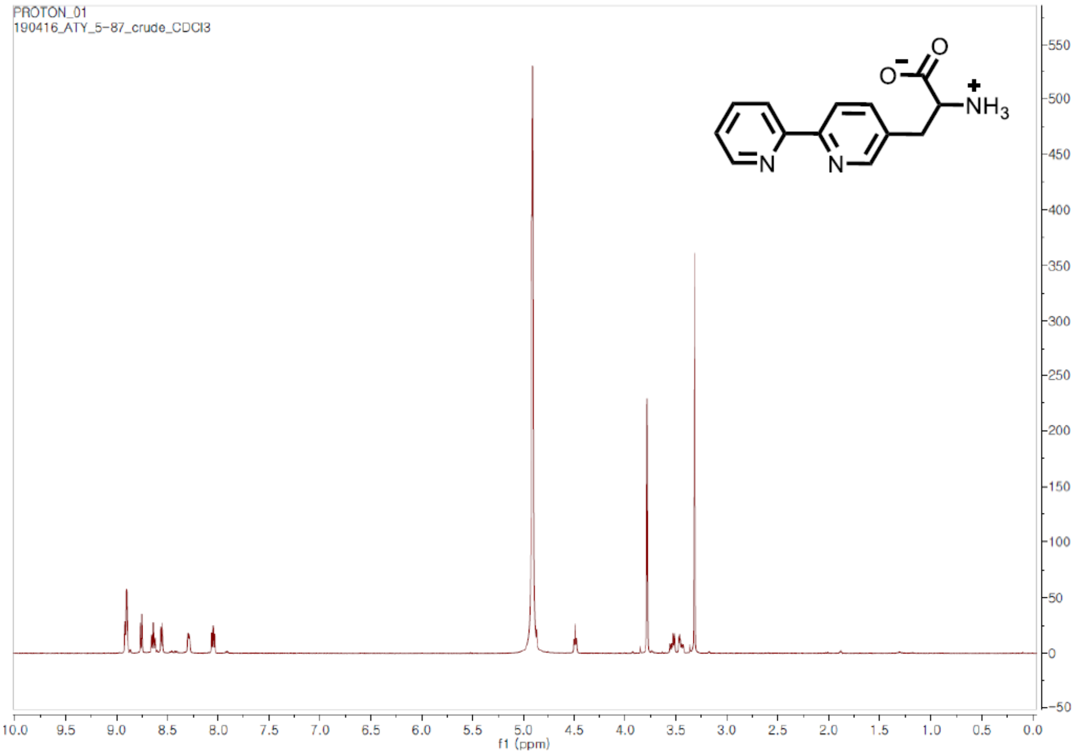

Supplementary Figure 5. NMR spectrum of Bpy-Ala **(6)**

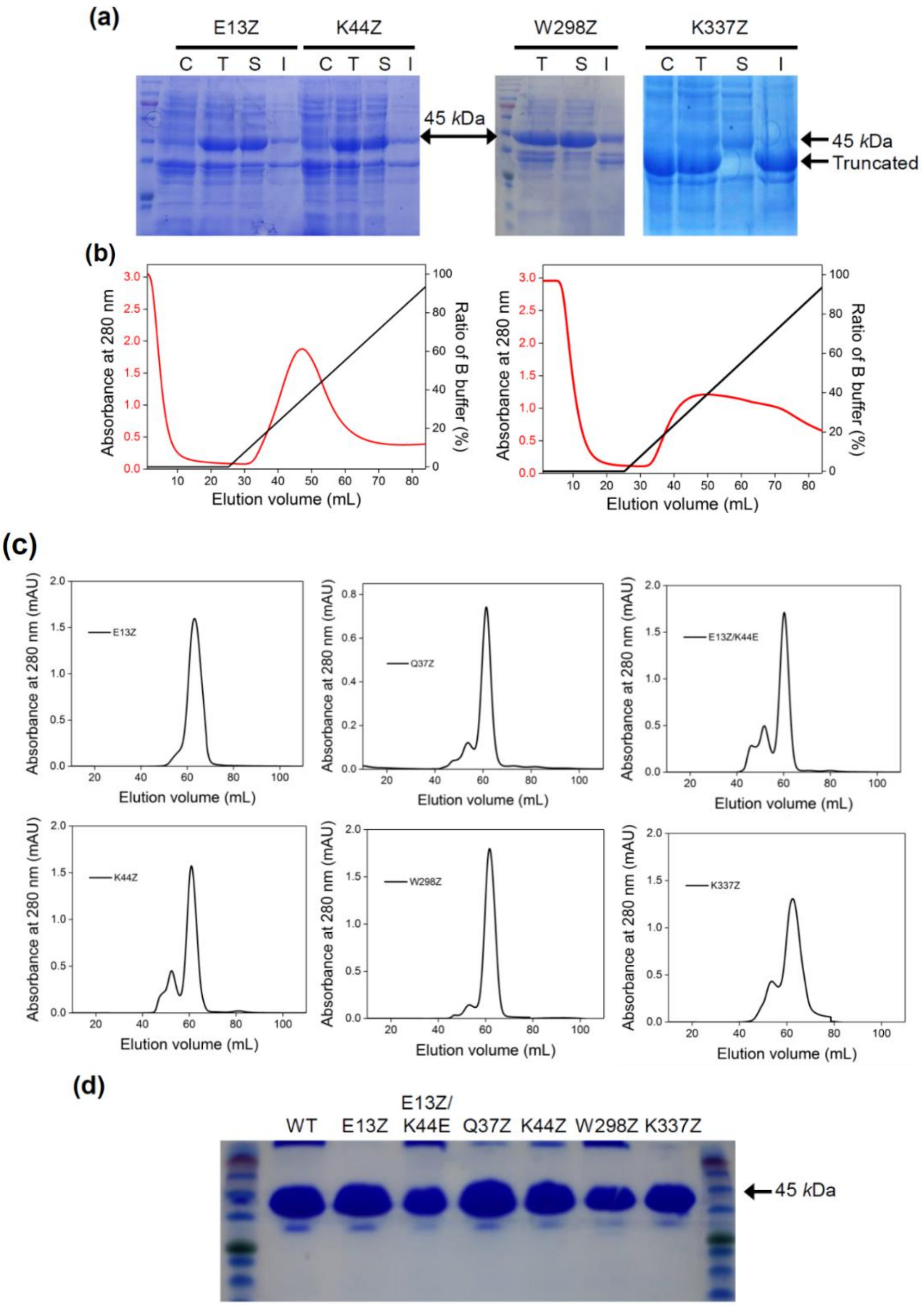

Supplementary Figure 6. Expression and purification of bpy-Ala variants. (a) 15% SDS-PAGE analysis of protein expression. C, T, S, and I indicate whole cells, lysates, soluble fractions, and inclusion body, respectively. (b) Purification of the bpy-Ala variants with Ni-NTA column chromatography. Representative elution traces of (left) K44Z and (right) E13Z/K44E (c) Size-exclusion chromatography (S200) of the apo proteins treated with EDTA. The native hexameric protein was eluted at ~60 mL, and residual higher molecular species were observed due to the metal impurities. (d) SDS-PAGE of the purified samples. The raw data in (a) and (d) are provided as a Source Data file.

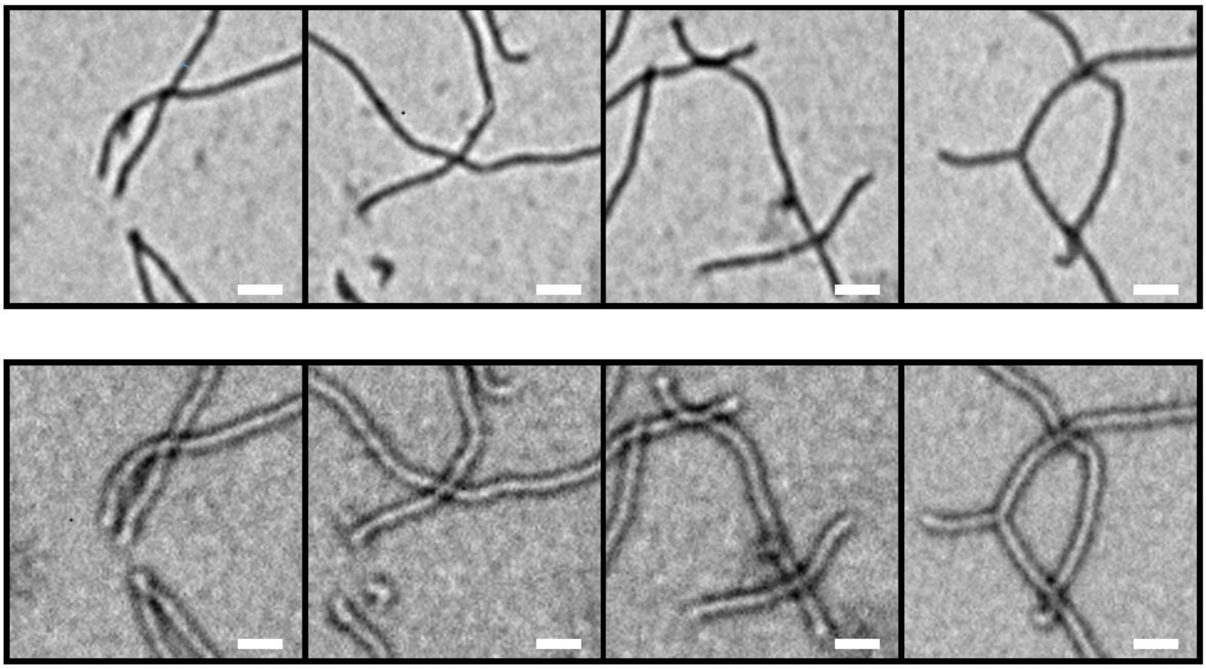

Supplementary Figure 7. TEM images of the linear rods collected with different focal points (scale bar: 50 nm)

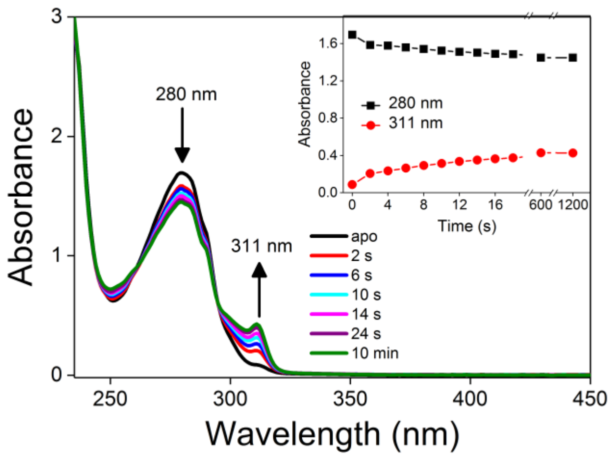

Supplementary Figure 8. Optical changes of K337Z protein (5 μM) upon the addition of Ni^{2+} (30 μM) at 25 °C (Inset: Time-dependent absorption changes at 280 and 311 nm). The raw data are provided as a Source Data file.

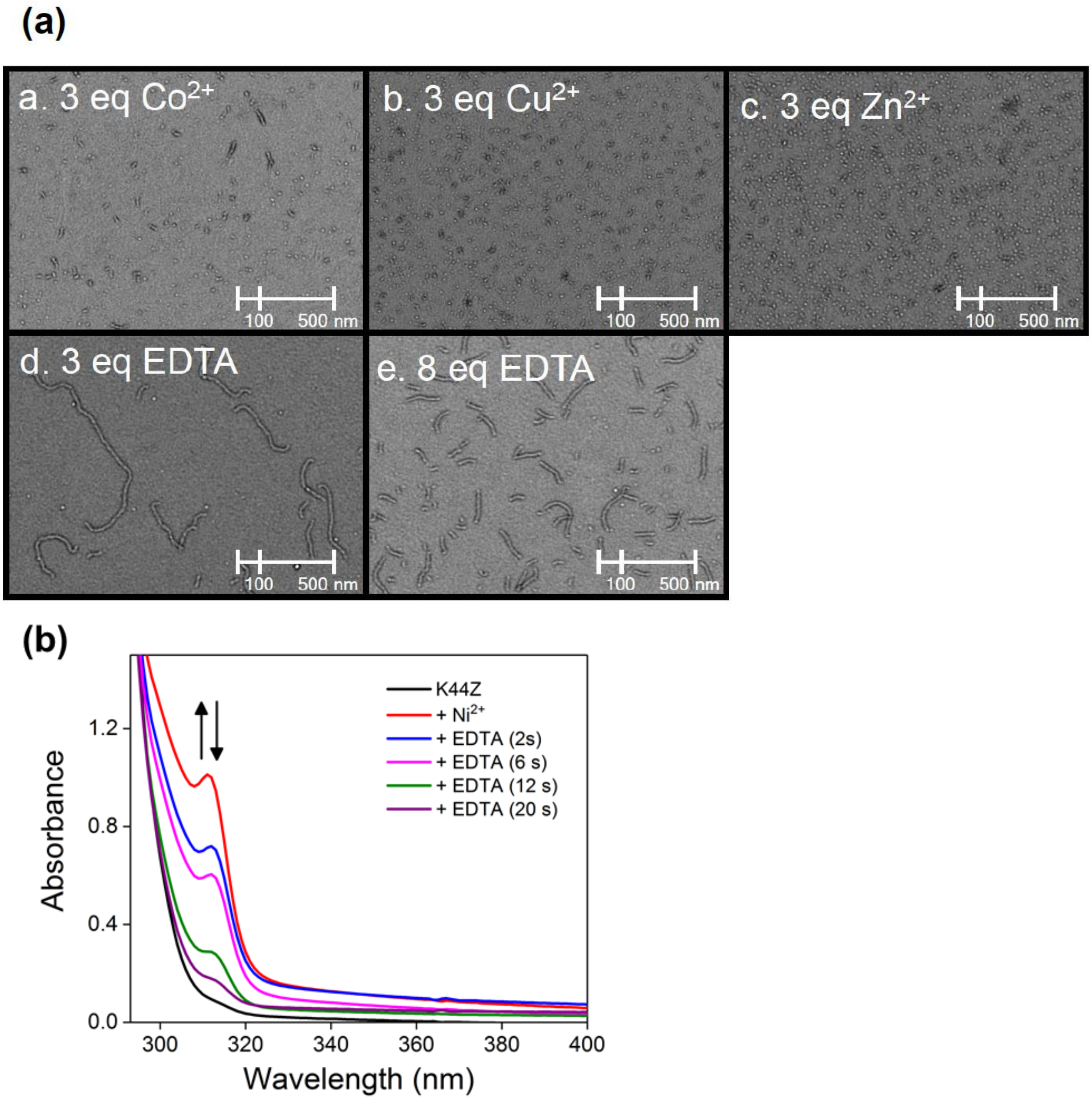

Supplementary Figure 9. Metal-dependent 1D-rod formation with K44Z. (a) TEM images of K44Z (10 μM) with various metal ions (3 equiv) at 22 °C for 24 h. Excess EDTA (3 or 8 equiv) was added to the pre-formed rod with Ni^{2+} for 24 h at 37 °C. (b) UV-Vis spectral changes of K44Z upon the addition of Ni^{2+} (3 equiv), followed by the introduction of EDTA (10^4-fold) at 25 °C. The raw data in Supplementary Figure 9b are provided as a Source Data file.

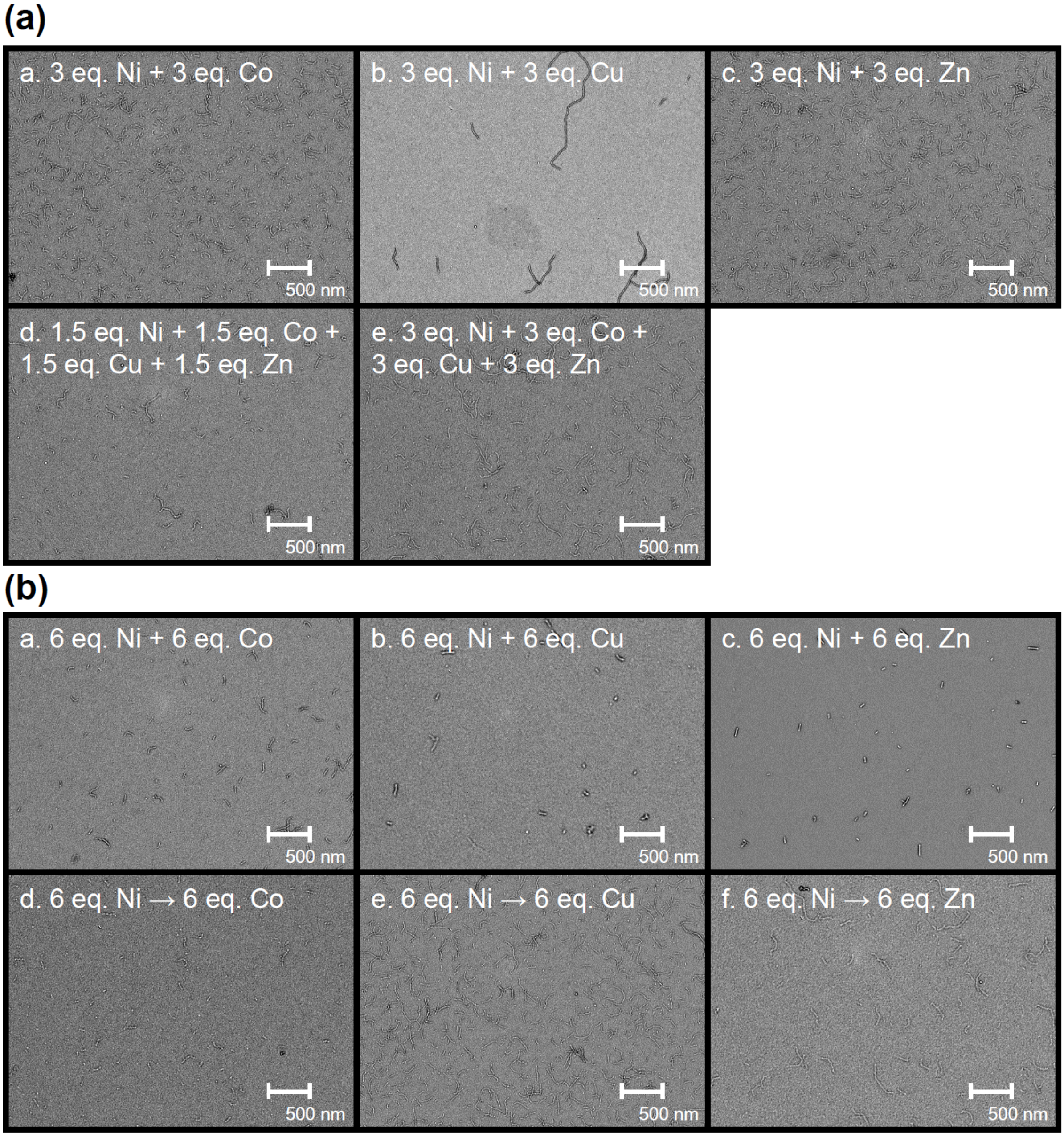

Supplementary Figure 10. TEM images of K44Z-linear assembly of with the mixtures of metal ions. (a) TEM images of K44Z (10 μM) incubated with more than one divalent metal ions together for 24 h at 37 ºC. (b) TEM images of K44Z (10 μM) upon the addition of metal ions in simultaneous (top) or sequential (bottom) manners

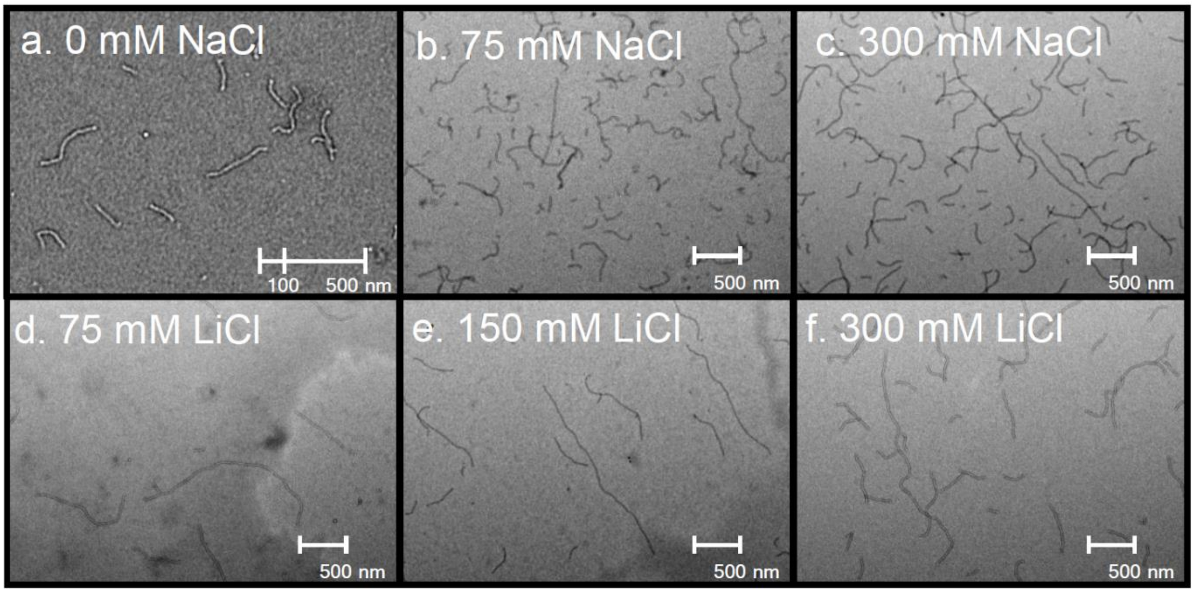

Supplementary Figure 11. TEM images of K44Z (10 μM) reacted with Ni^{2+} (a) 3 equiv Ni^{2+} at 22 ºC (b-f) 8 equiv Ni^{2+} at 37 ºC for 24 h with [NaCl] or [LiCl] = 0–300 mM

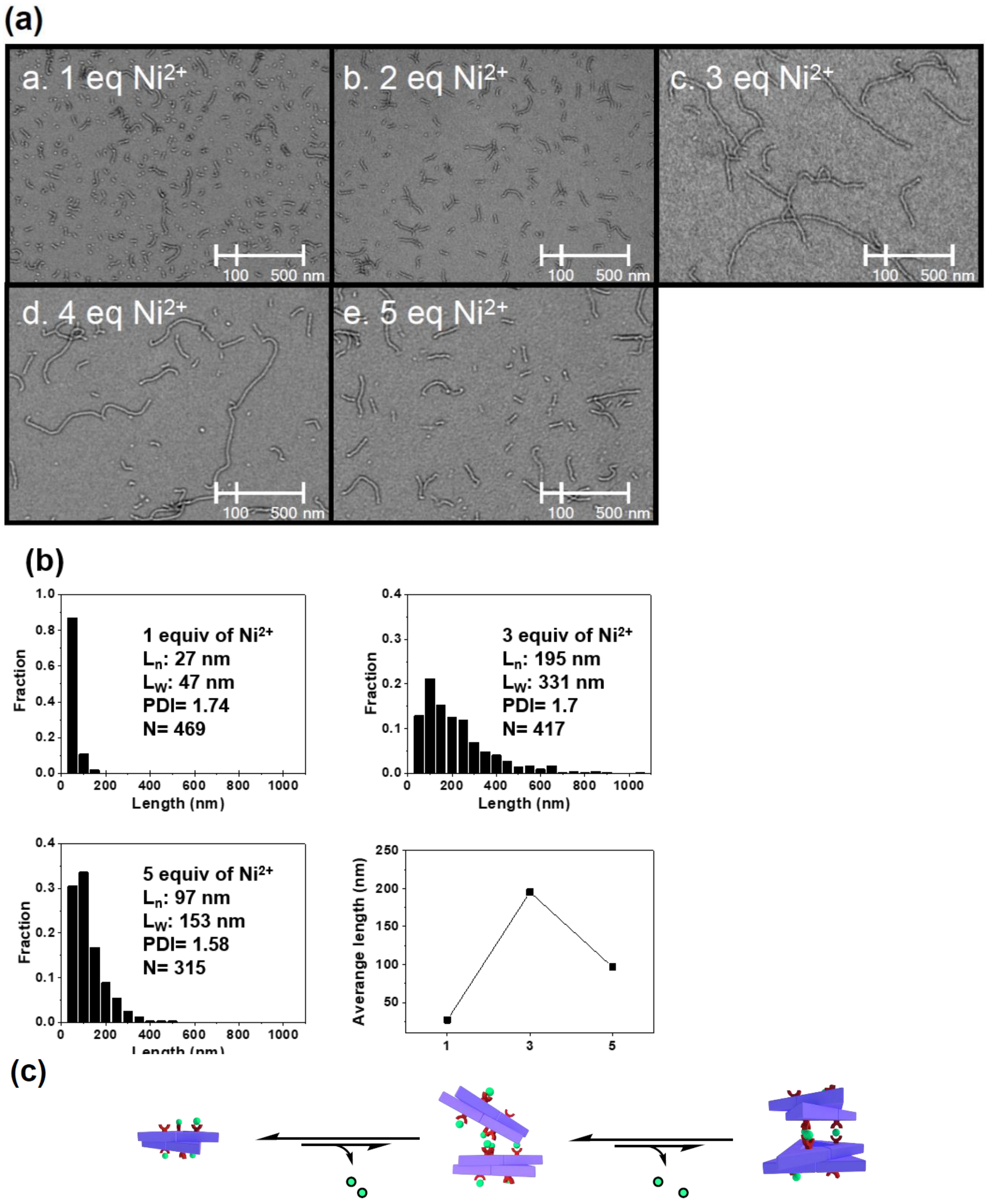

Supplementary Figure 12. K44Z (10 μM) with various ratios of Ni^{2+} at 4 °C for 24 h. (a) TEM images (b) Distributions of the 1D-rods in (a) and the number average length (L_n) of the rods as a function of Ni^{2+} to protein ratio. The L_n, L_w, PDI, and N values indicate number average length, weight average length, polydispersity index, and the number of counted 1D-assembled rods, respectively. The 1D-rods were maximized at 4 °C with 3 equiv of Ni^{2+} to protein. (c) A proposed scheme for the 1D self-assembly. The raw data in Supplementary Figure 12b are provided as a Source Data file.

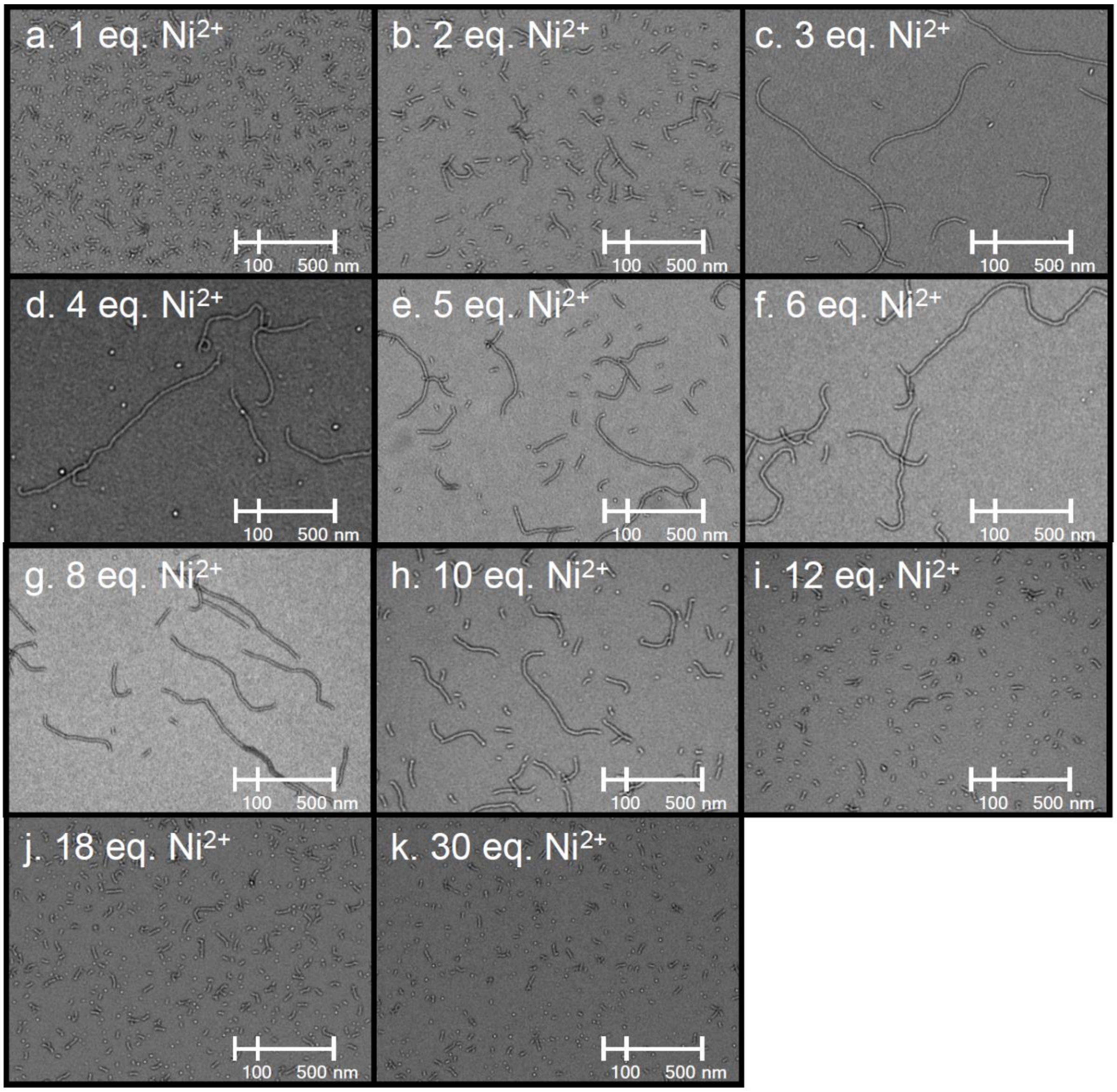

Supplementary Figure 13. TEM images of 1D-rods formed with K44Z protein (10 μM) and (a) 1 (b) 2 (c) 3 (d) 4 (e) 5 (f) 6 (g) 8 (h) 10 (i) 12 (j) 18 (k) 30 equiv of Ni^{2+} at 22 ºC. The longest rod-shaped materials were observed when 3−6 equiv of Ni^{2+} to 1 equiv of protein was added.

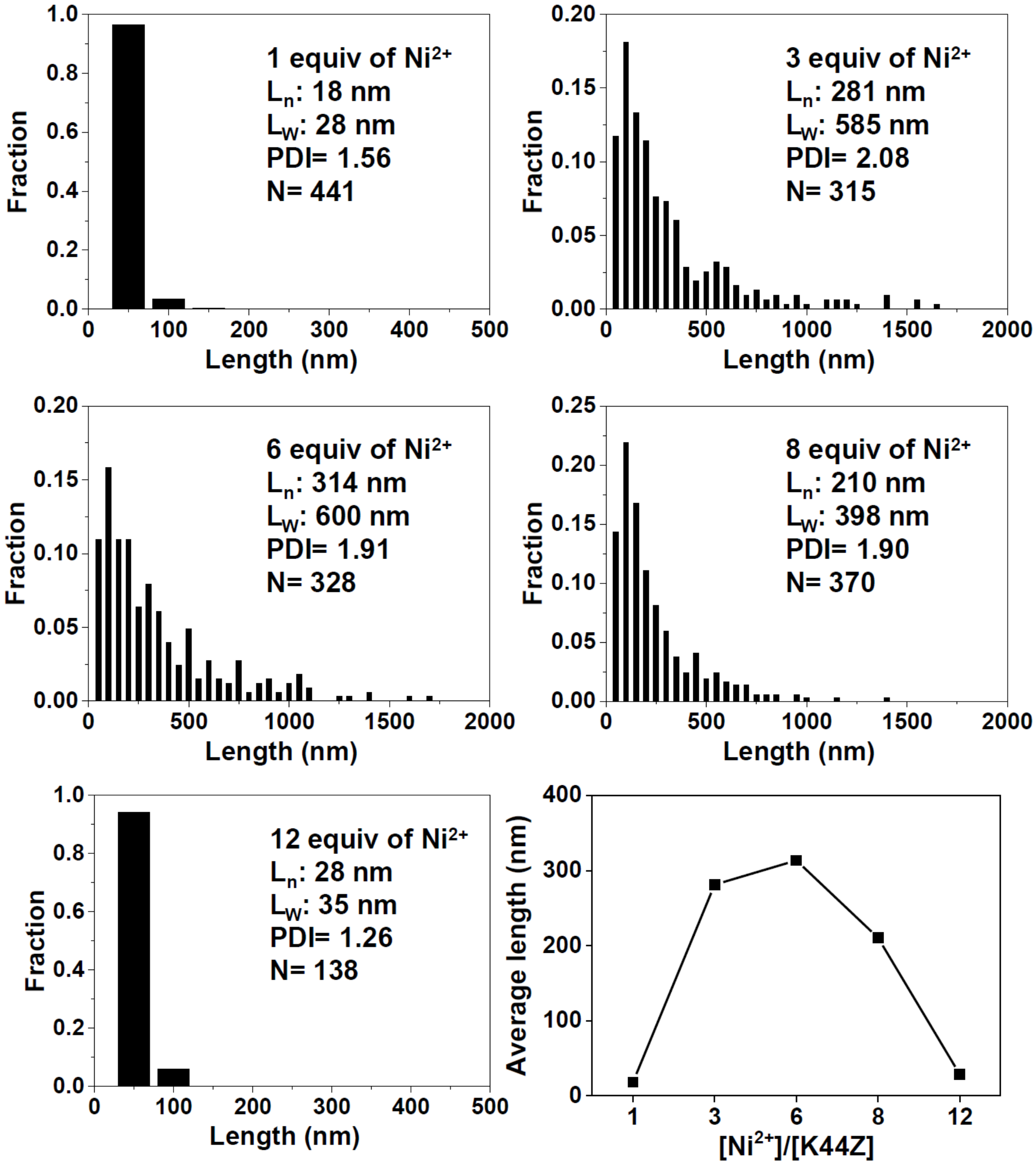

Supplementary Figure 14. Distributions of 1D rods shown in Supplementary 13 and the average length (L_n) of the rods as a function of Ni^{2+} to protein ratio. The L_n, L_w, PDI, and N values indicate number average length, weight average length, polydispersity index, and the number of counted 1D-assembled rods, respectively. The optimal ratio of metal to protein was determined to be 3–6 equiv at 22 °C. The raw data in Supplementary Figure 14 are provided as a Source Data file.

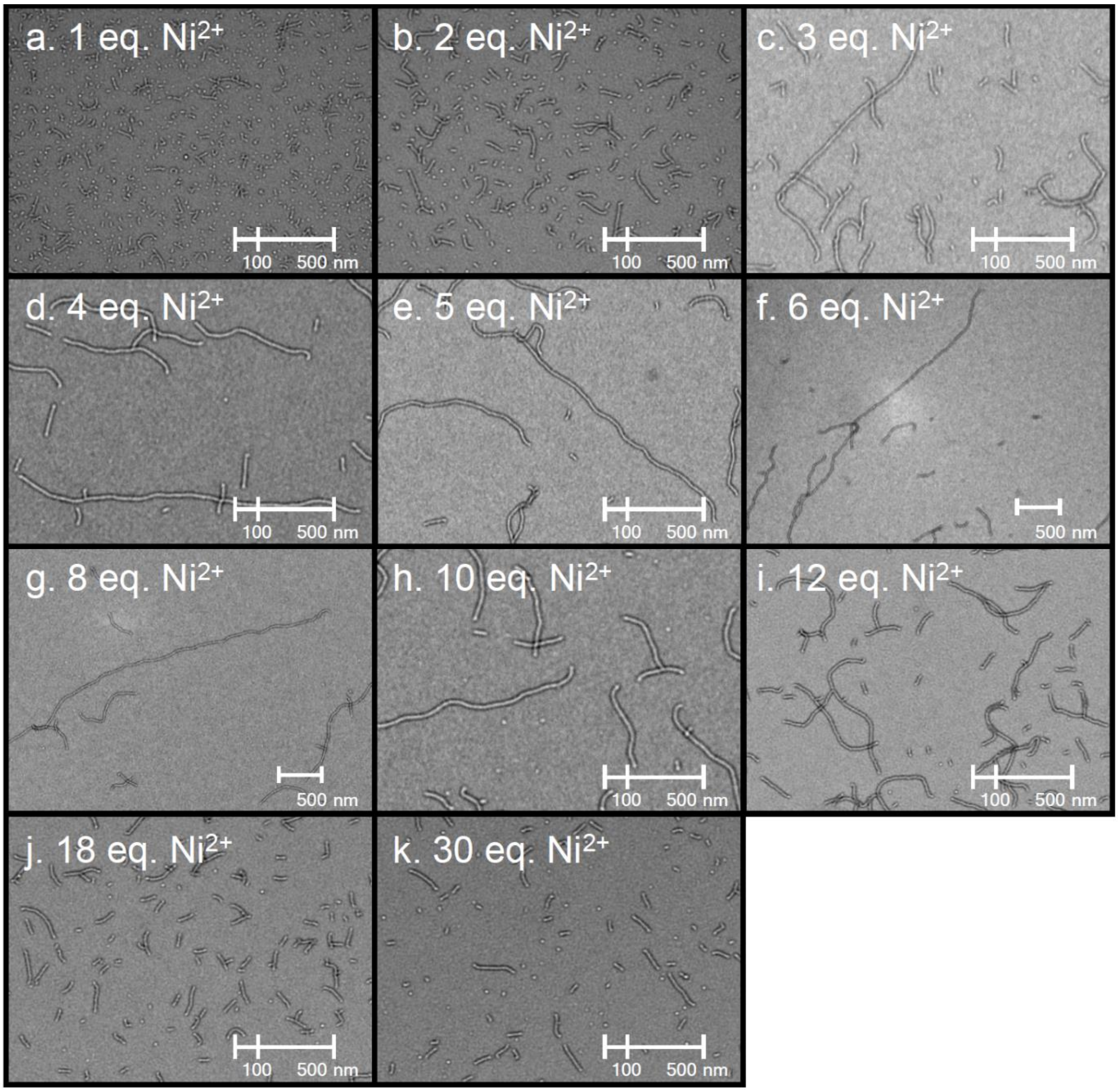

Supplementary Figure 15. TEM images of 1D rods formed upon the addition of various concentrations of Ni^{2+} to K44Z protein (10 μM) at 37 ºC. (a) 1 (b) 2 (c) 3 (d) 4 (e) 5 (f) 6 (g) 8 (h) 10 (i) 12 (j) 18 (k) 30 equiv of Ni^{2+}. The optimized ratio of metal to protein was determined to be 8 equiv of Ni^{2+} at 37 ºC, higher than the values observed at lower temperatures.

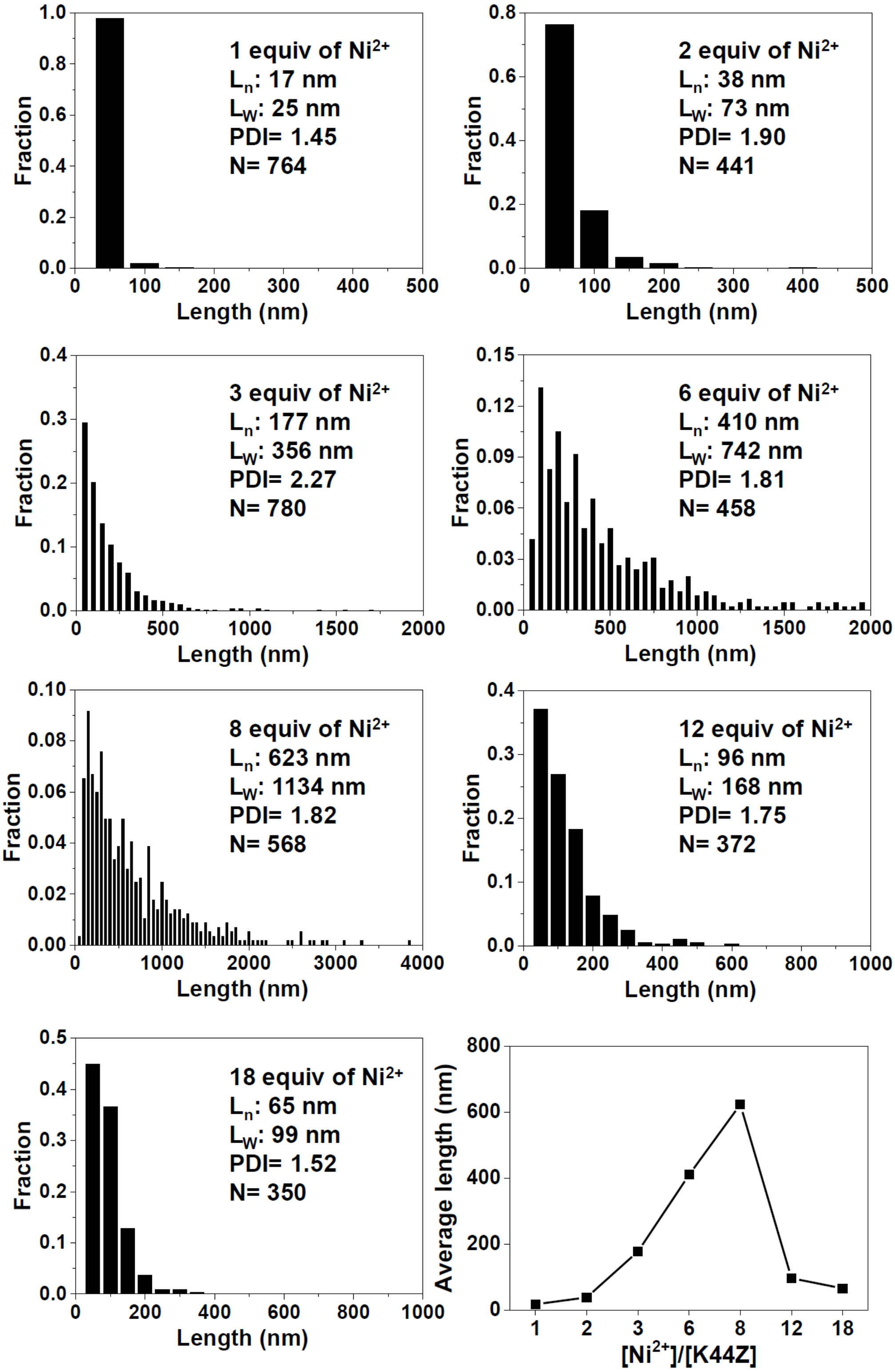

Supplementary Figure 16. Distributions of K44Z (10 µM) 1D-rods assembled at 37 ºC and the average length (L_n) of the rods as a function of Ni^{2+} to protein ratio. The L_n, L_w, PDI, and N values indicate number average length, weight average length, polydispersity index, and the number of counted 1D-assembled rods, respectively. The optimal ratio of metal to protein is 8 at 37 ºC. The raw data in Supplementary Figure 16 are provided as a Source Data file.

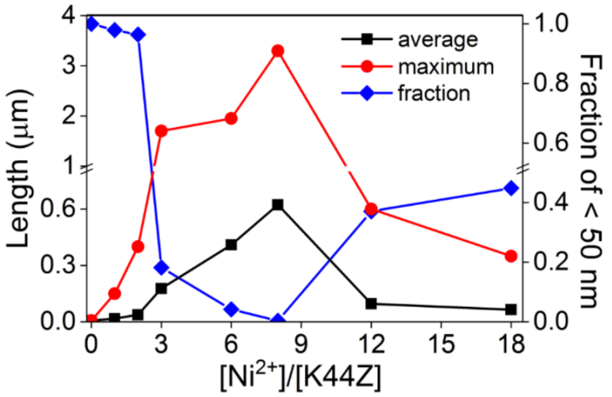

Supplementary Figure 17. Growth of 1D rods of K44Z (10 μM) at 37 °C by varying the ratios of Ni^{2+} to protein. The fraction of less than 50 nm protein species was consumed as the rods grow. The raw data in Supplementary Figure 17 are provided as a Source Data file.

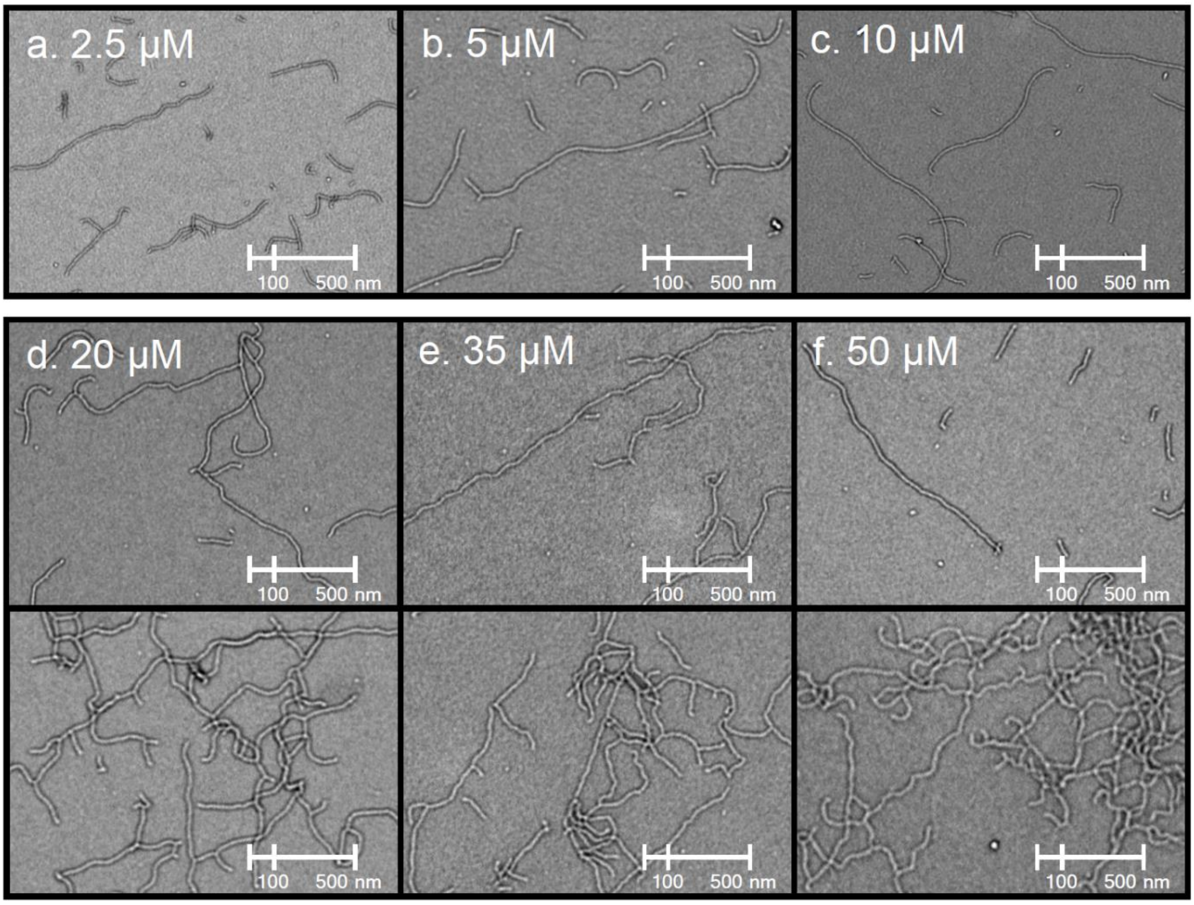

Supplementary Figure 18. TEM images of rods formation with various concentrations of K44Z protein with the optimal ratios of Ni^{2+} (3 equiv) at 22 ºC. (a) 2.5 μM (b) 5 μM (c) 10 μM (d) 20 μM (e) 35 μM (f) 50 μM of K44Z. Upon the variation of protein concentrations, no considerable alteration in the length of the 1D rods was observed. Instead, the relative distributions of the entangled 1D rods become more dominant when higher protein concentration (20–50 μM) was applied (bottoms in d, e, and f).

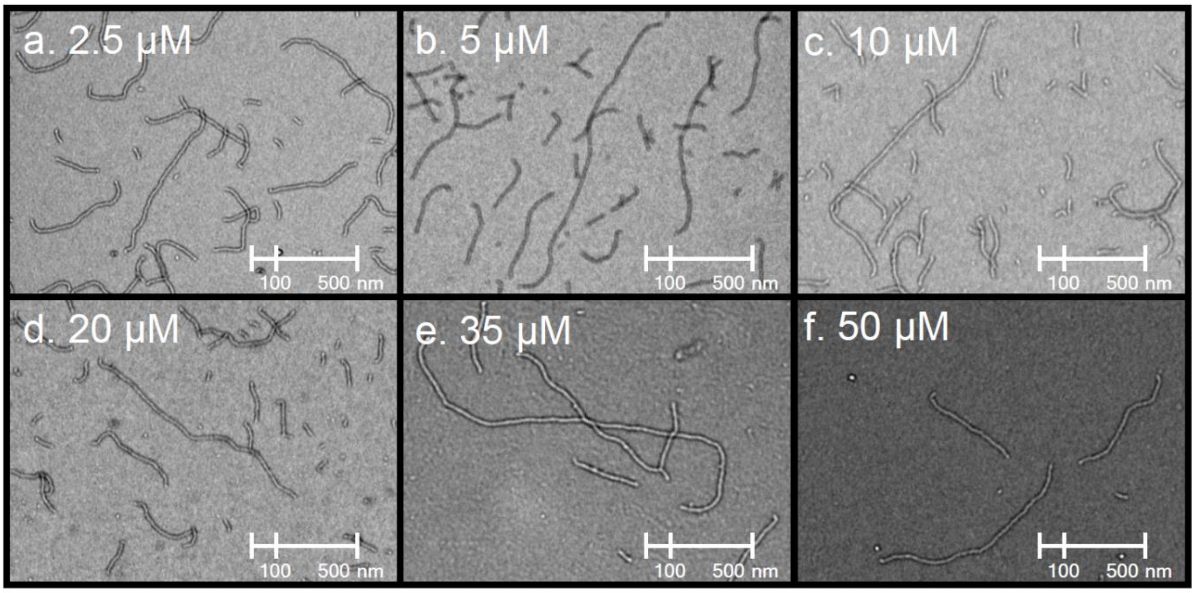

Supplementary Figure 19. TEM images of the 1D rods with various concentrations of K44 proteins with 3 equiv Ni^{2+} at 37 ºC. (a) 2.5 μM (b) 5 μM (c) 10 μM (d) 20 μM (e) 35 μM (f) 50 μM of K44Z protein. No considerable difference in the length of the protein rods was observed upon the variations of the protein concentrations.

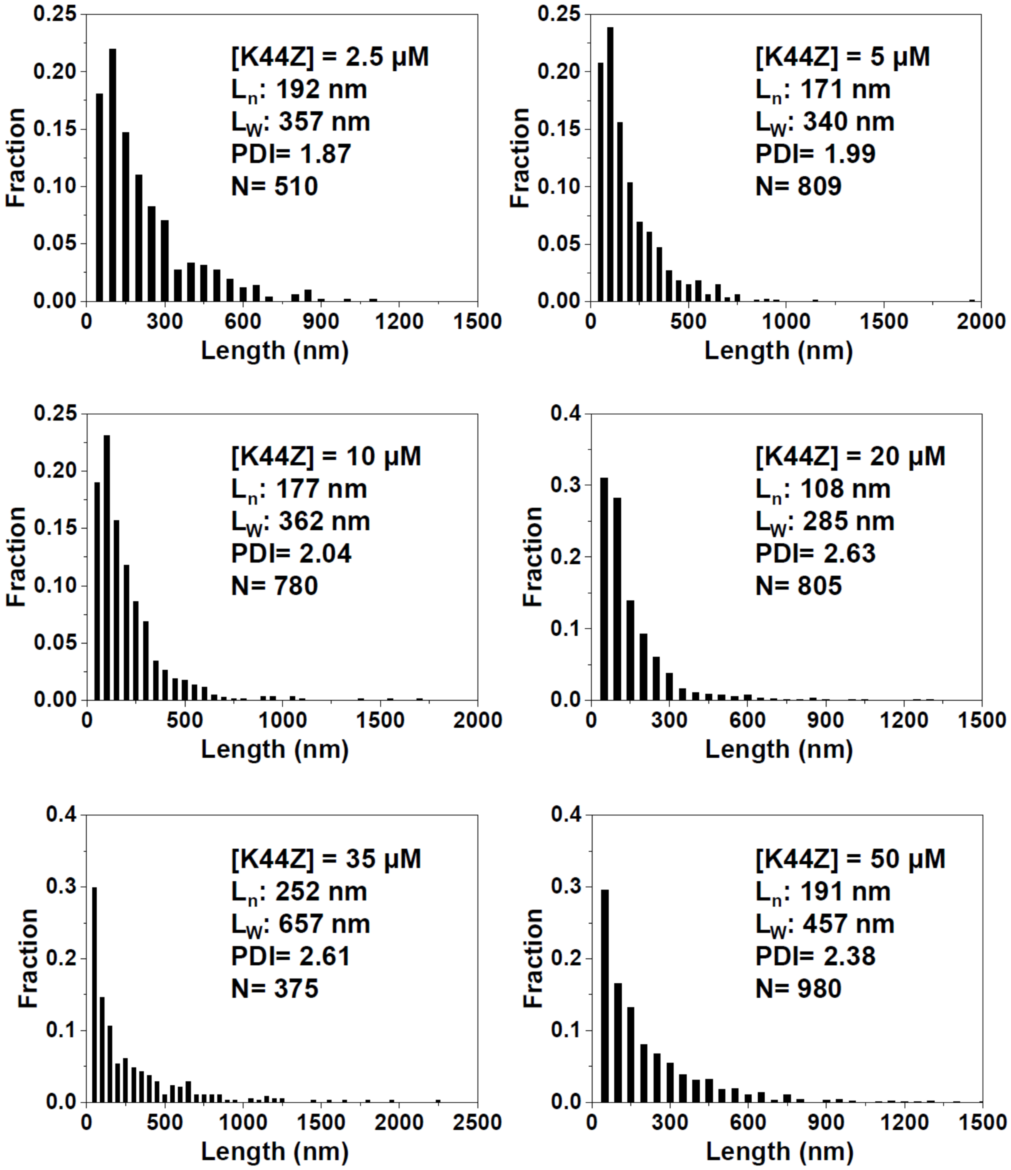

Supplementary Figure 20. Distributions of K44Z-derived 1D-rods with various concentrations of K44Z (2.5–50 μM) and 3 equiv of Ni^{2+} at 37 ºC for 24 h. The L_n, L_w, PDI, and N values for each histogram indicate number average length, weight average length, polydispersity index, and the number of counted 1D-assembled rods, respectively. No considerable difference in the length of the protein rods was observed upon the variations of the protein concentrations. The raw data in Supplementary Figure 20 are provided as a Source Data file.

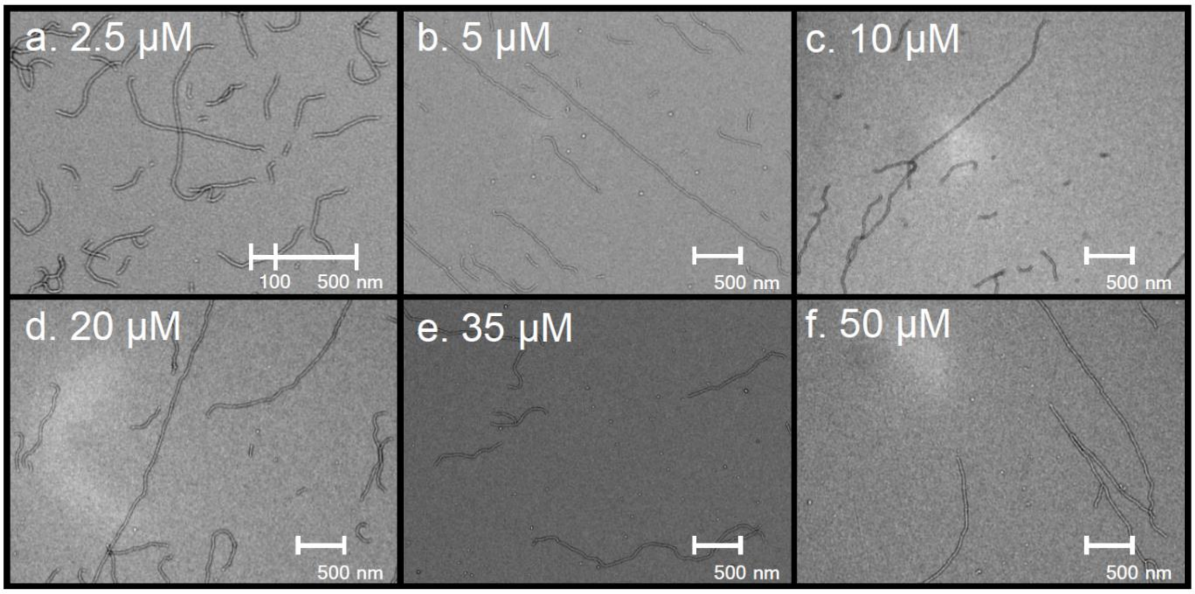

Supplementary Figure 21. TEM images of the 1D rods formed with the various concentrations of K44Z protein and 6 equiv of Ni^{2+} at 37 ºC for 24 h. (a) 2.5 µM (b) 5 µM (c) 10 µM (d) 20 µM (e) 35 µM (f) 50 µM of K44Z. With the increasing protein concentrations, longer 1D rods were formed. The results contrast to those in Supplementary Figures 18 and 19, when 3 equiv of Ni^{2+} was reacted at 22 ºC and 37 ºC.

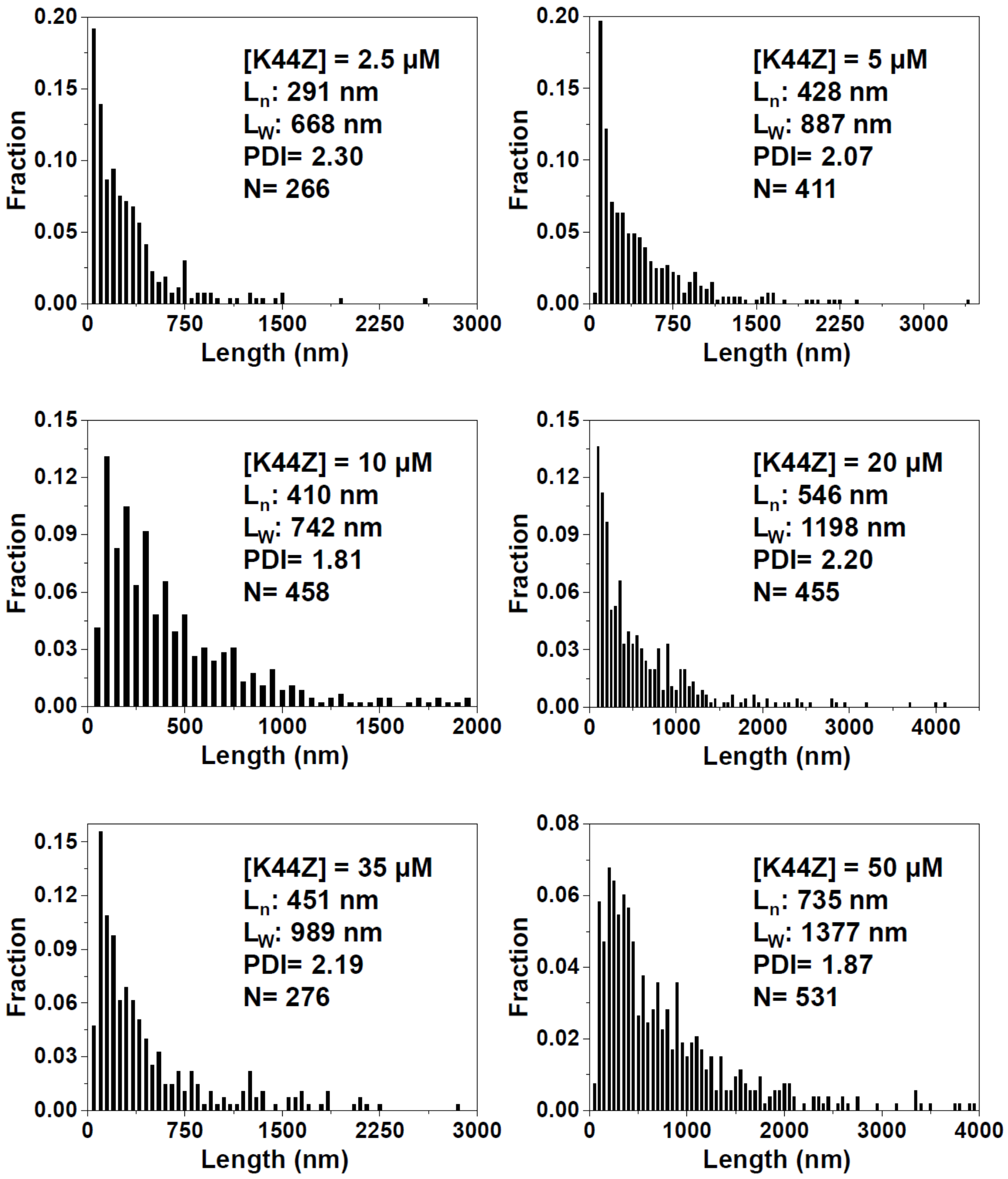

Supplementary Figure 22. Distributions of the 1D-rods formed with K44Z (2.5–50 µM) and 6 equiv. (the less than optimal) of Ni^{2+} at 37 ºC. The L_n, L_w, PDI, and N values for each histogram indicate number average length, weight average length, polydispersity index, and the number of counted 1D-assembled rods, respectively. With the increasing protein concentrations, longer 1D rods were formed. The raw data in Supplementary Figure 22 are provided as a Source Data file.

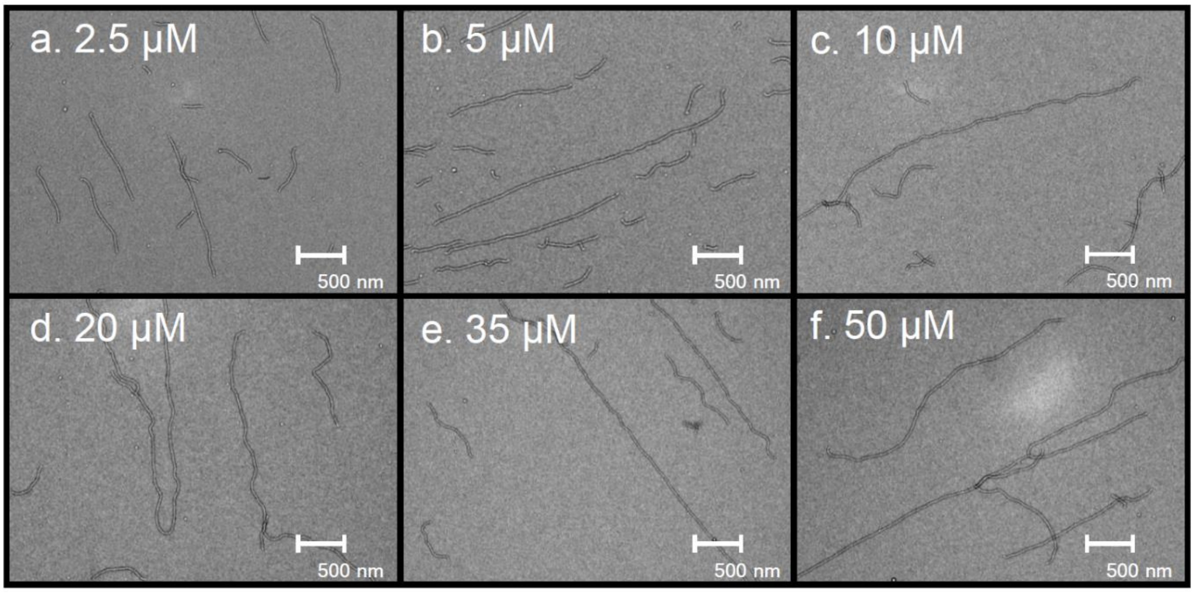

Supplementary Figure 23. TEM images of 1D rods from the reactions of various concentrations of K44Z and the optimal ratio of Ni^{2+} (8 equiv.) at 37 ºC. (a) 2.5 μM (b) 5 μM (c) 10 μM (d) 20 μM (e) 35 μM (f) 50 μM of K44 protein. Similar to the data in Supplementary Figure 21, logner protein rods were formed with the higher concentrations of proteins.

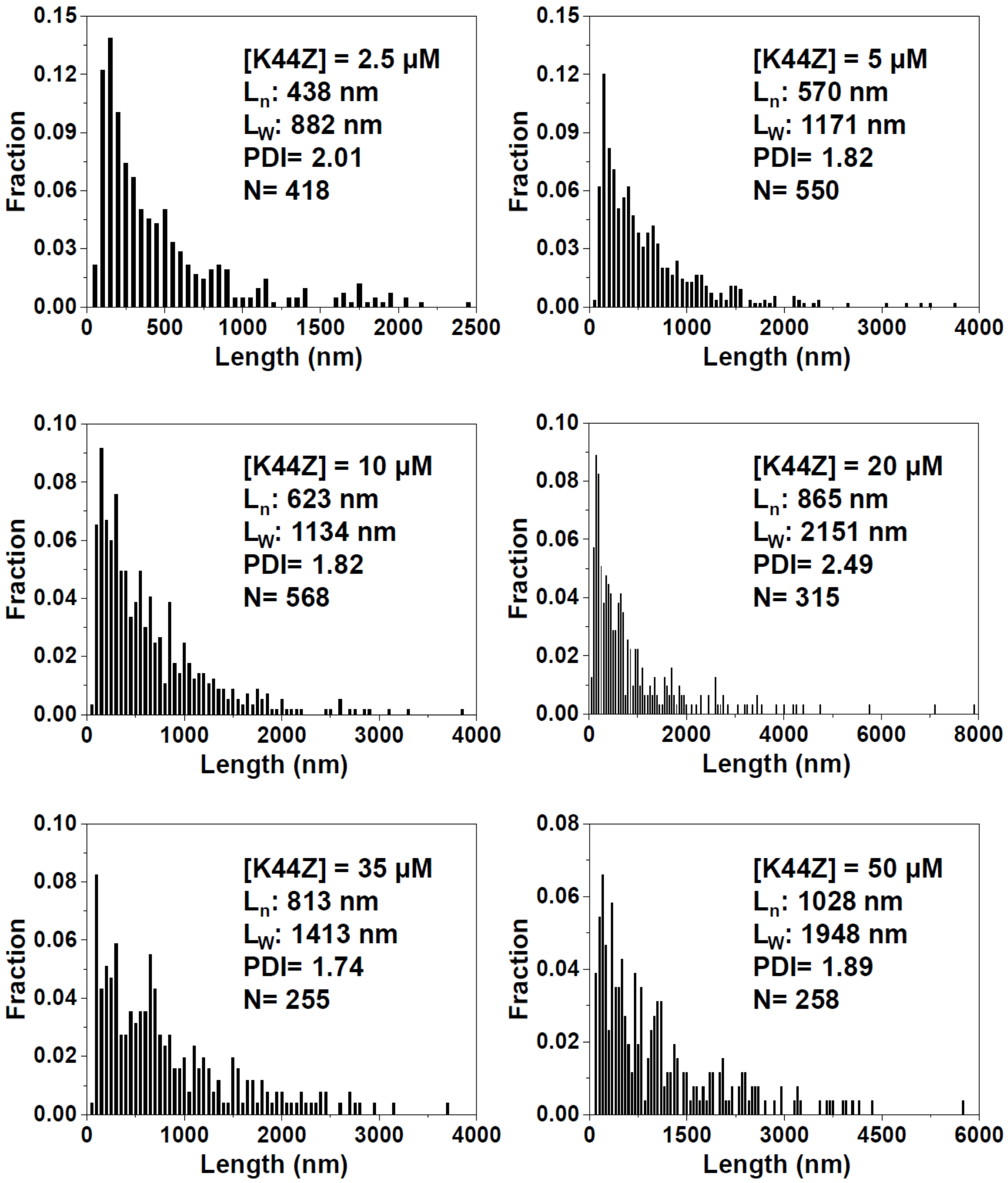

Supplementary Figure 24. Distributions of 1D-rods from Supplementary Figure 23. The L_n, L_w, PDI, and N values indicate number average length, weight average length, polydispersity index, and the number of counted 1D-assembled rods, respectively. With the increasing protein concentrations, longer 1D rods were formed. The raw data in Supplementary Figure 24 are provided as a Source Data file.

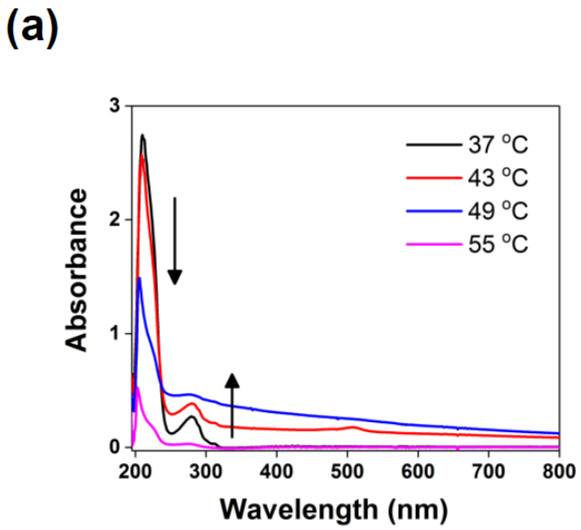

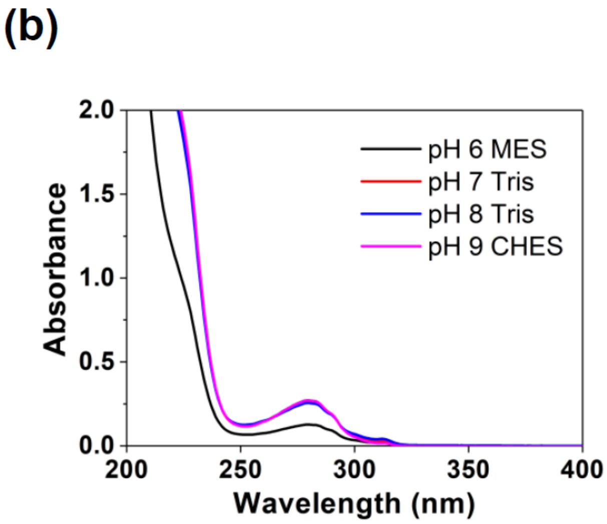

Supplementary Figure 25. Optical spectral changes of K44Z (10 μM) under various conditions. (a) UV-Vis spectra of the protein incubated at 37–55 ºC for 24 h in 50 mM Tris, pH 7 buffer. (b) UV-Vis spectra change of the protein incubated at 37 ºC after buffer exchange with 50 mM buffer with various pH 6–9 ranges. The samples were centrifuged, and the supernatants were applied for detction. K44Z was stable only up to 37 ºC and pH 7–9 values. The raw data in Supplementary Figure 25 are provided as a Source Data file.

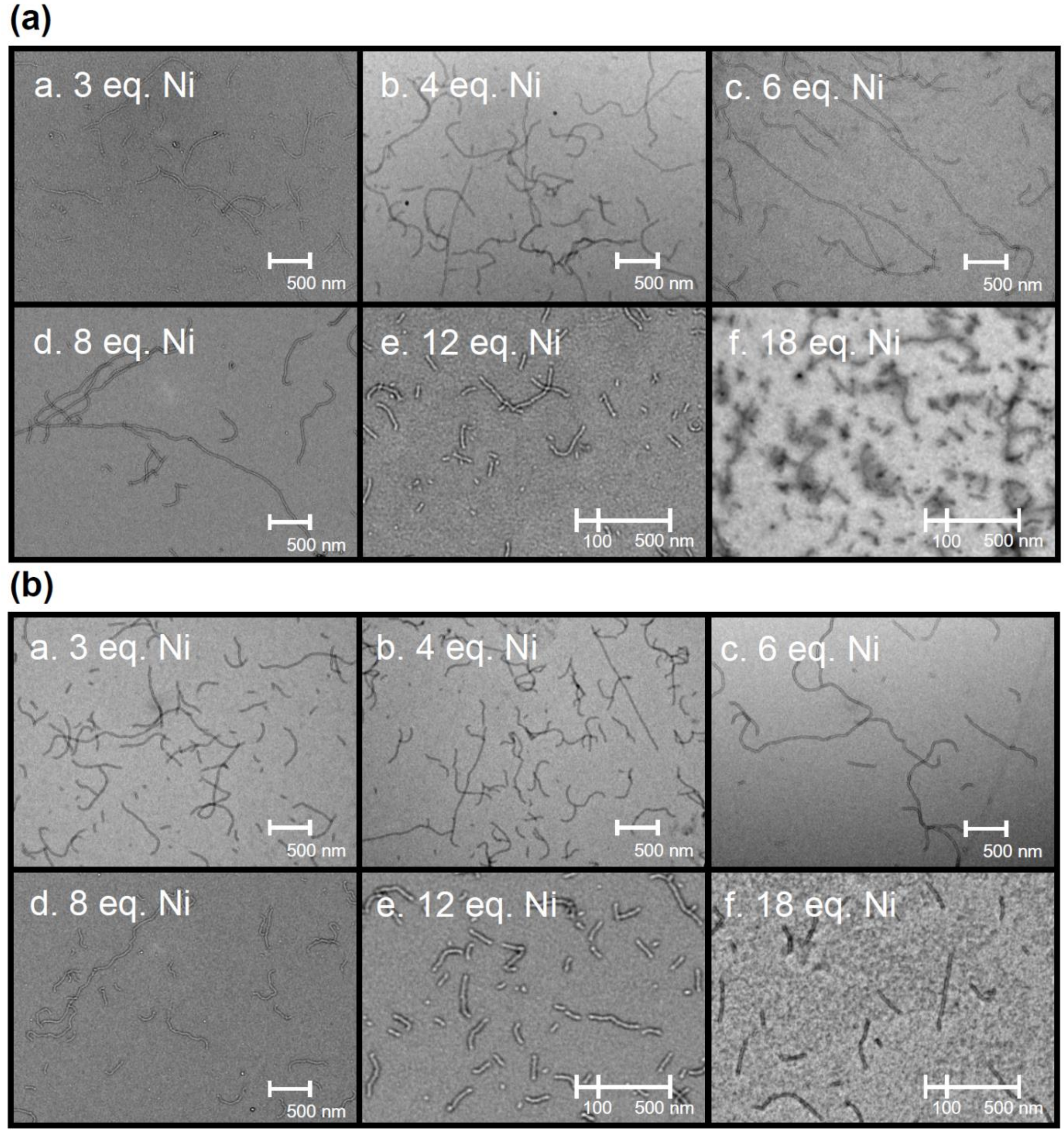

Supplementary Figure 26. Formation and stability of K44Z-derived 1D-rods at various temperatures. K44Z (10 μM) were incubated with various concentraitons of Ni^{2+} at (a) 43 °C and (b) 49 °C. No rod formation was observed at 55 ^{o}C (not shown). The optimal ratios at 43 and 49 ^{o}C were determined to be 6 equiv. Ni^{2+} at both conditions, which is lower than the value at 37 ^{o}C (8 equiv.). Concentrations larger than 8 equiv. of metal ions at 43 and 49 ^{o}C yielded precipitants.

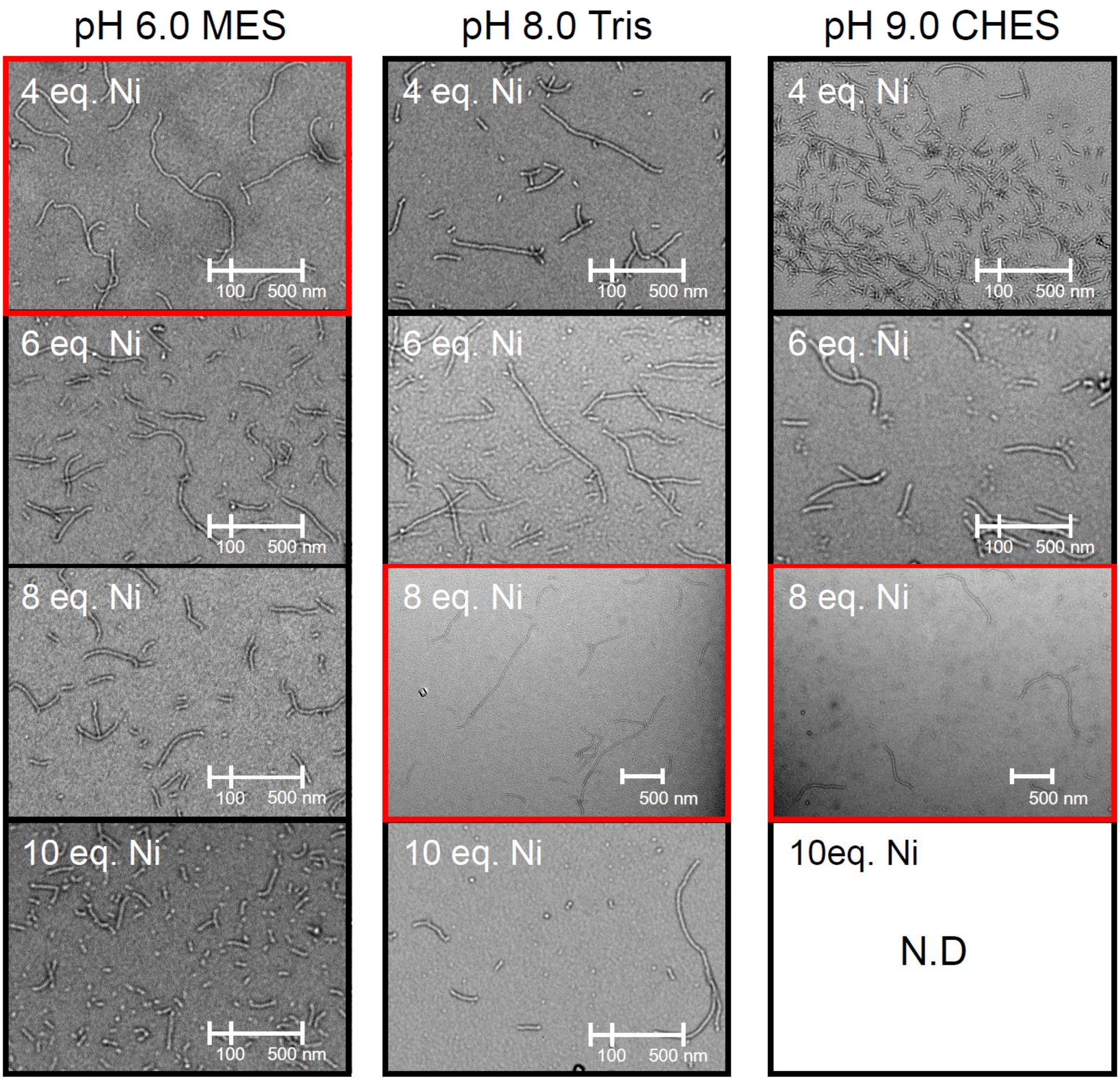

Supplementary Figure 27. Formation of K44Z-derived rods at various pH condition. K44Z (7.5 μM) and the various concentrations of Ni^{2+} were incubated in pH 6–9 buffers (50 mM MES pH 6, 50 mM Tris at 7–8, 50 mM CHES pH 9) at 37 °C. The optimal ratios of Ni^{2+} to the protein were marked with red boxes for each pH condition.

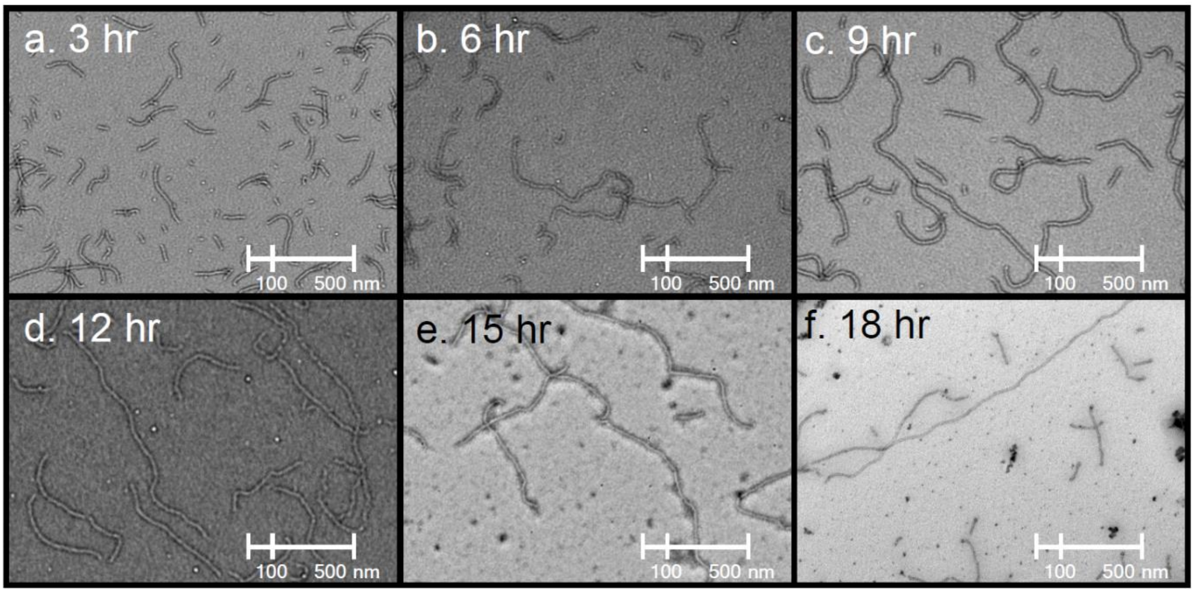

Supplementary Figure 28. Formation kinetics of 1D rod formation with K44Z protein with 6 equiv Ni^{2+}. TEM images were acquired after the incubation of of K44Z (5 μM) with 6 equiv Ni^{2+} at 37 ºC for (a) 3 h (b) 6 h (c) 9 h (d) 12 h (e) 15 h (f) 18 h. Protein rods with various lengths were observed, implying that 1D assembly of K44Z proceeds via step-growth polymerization mechanism.

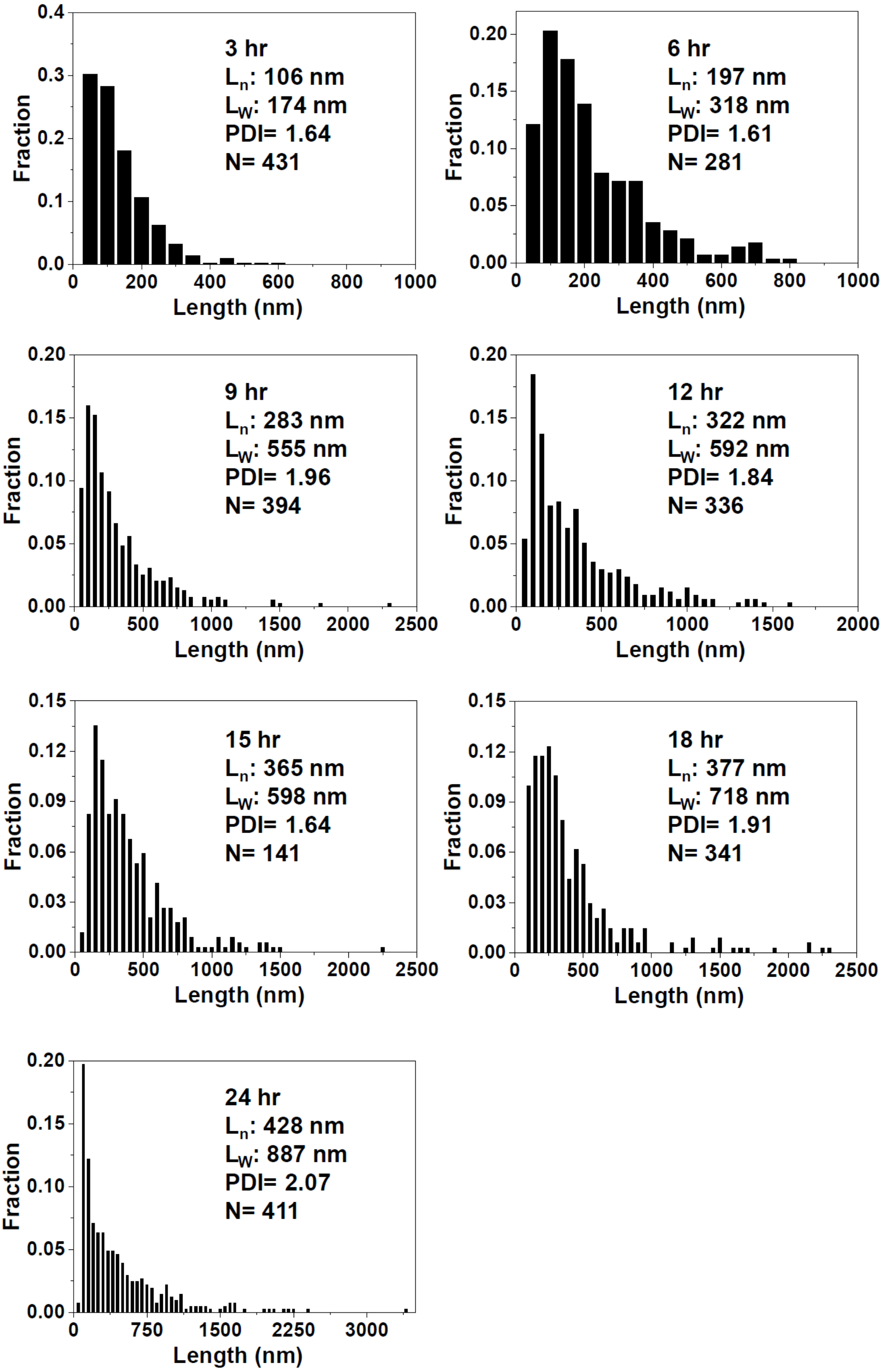

Supplementary Figure 29. Time-dependent length changes determined from the TEM images in Supplementary Figure 28. The L_n, L_w, PDI, and N values indicate number average length, weight average length, polydispersity index, and the number of counted 1D-assembled rods, respectively. The raw data in Supplementary Figure 29 are provided as a Source Data file.

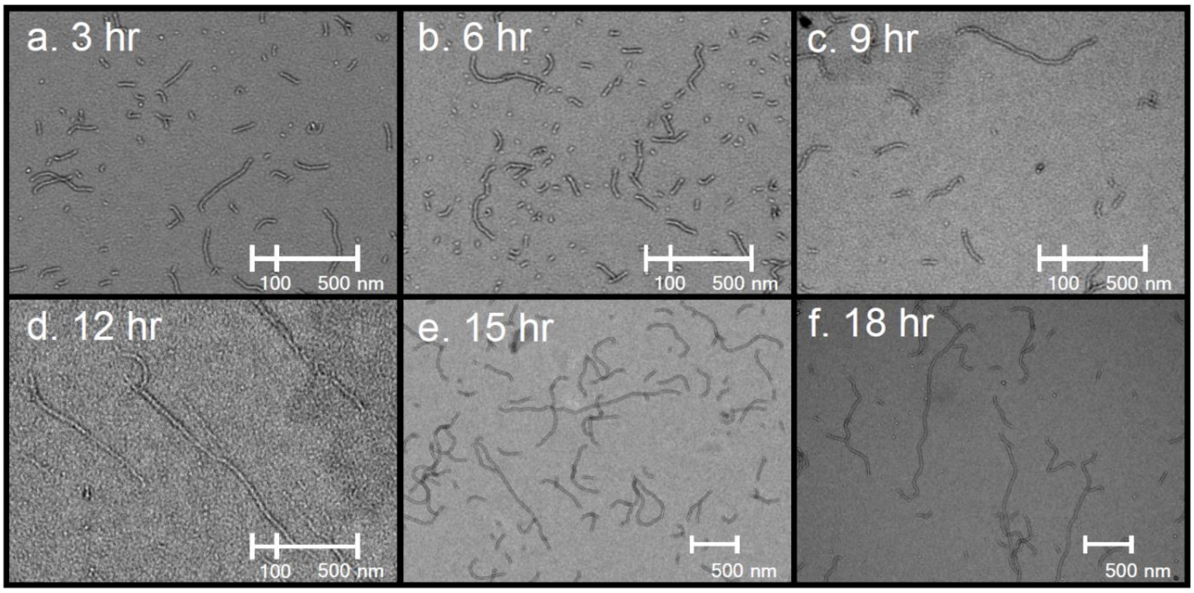

Supplementary Figure 30. Formation kinetics of 1D rod formation with K44Z protein with 8 equiv. Ni^{2+}. TEM images were acquired after the incubation of K44Z (5 μM) with 8 equiv Ni^{2+} at 37 ºC for (a) 3 h (b) 6 h (c) 9 h (d) 12 h (e) 15 h (f) 18 h. Similar to the data in Supplementary Figure 28, the protein rods with various lengths were observed, indicating that the step-growth polymerization mechanism is operative.

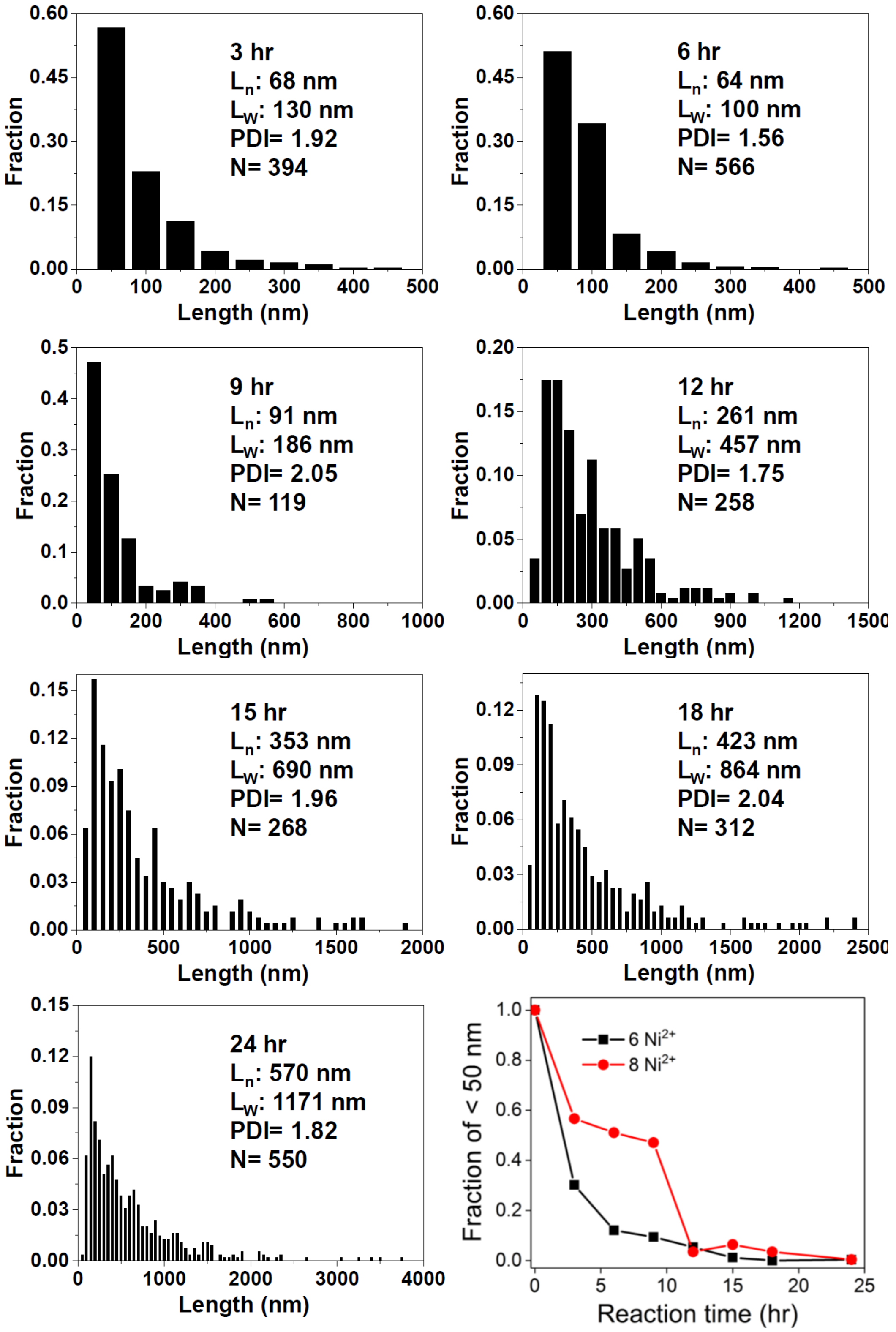

Supplementary Figure 31. Time-dependent distributions of 1D-rods shown in Supplementary Figure 30 and the average length (L_n) as a function of reaction time. The L_n, L_w, PDI, and N values indicate number average length, weight average length, polydispersity index, and the number of counted 1D-assembled rods, respectively. The raw data in Supplementary Figure 31 are provided as a Source Data file.

(a)

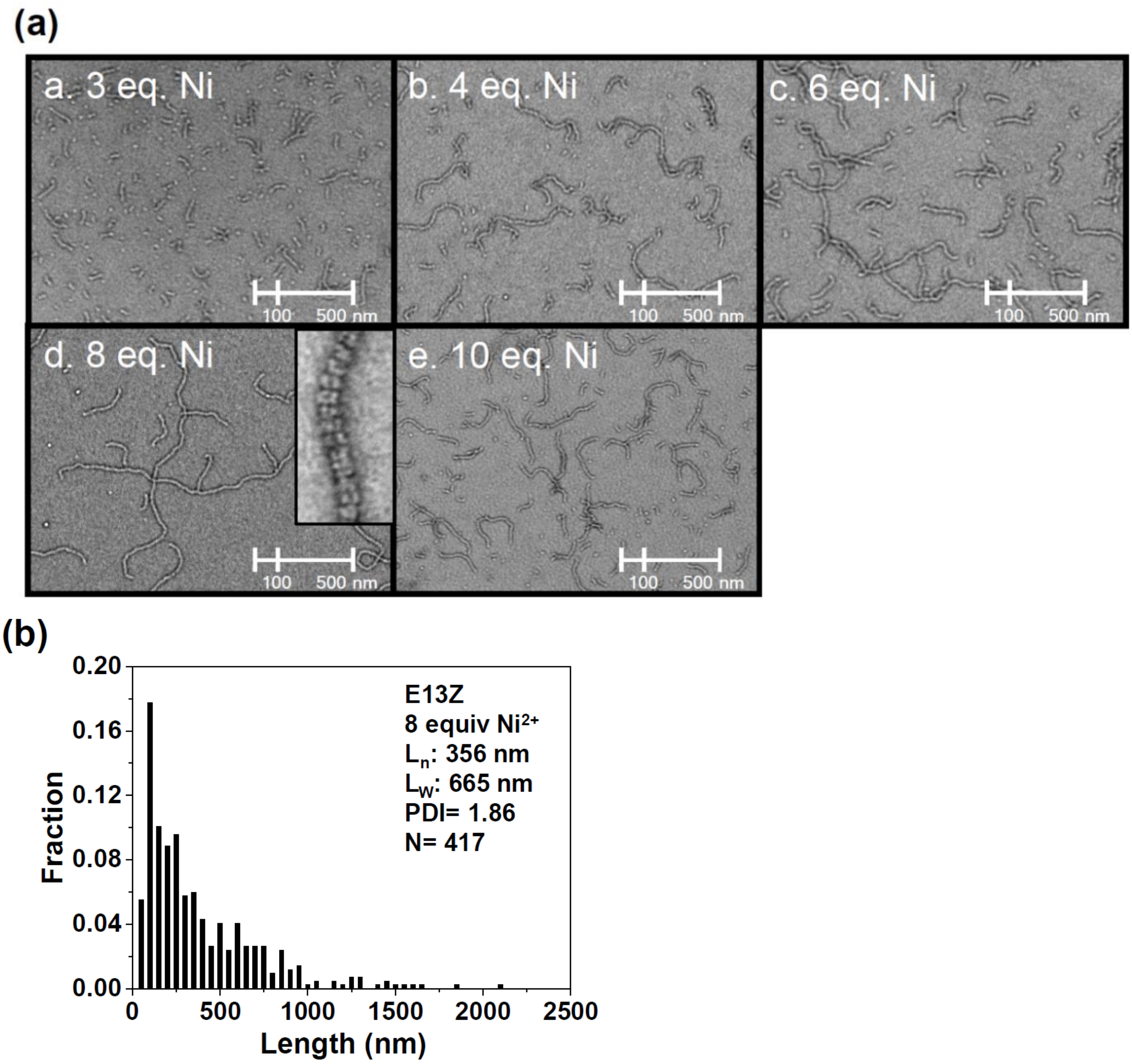

Supplementary Figure 32. Formation of 1D-rods with E13Z and Ni^{2+}. (a) TEM images of 1D-rods from the reactions of E13Z (10 μM) and Ni^{2+} (3–10 equiv) at 37 ºC. (b) Distribution of the number average length when the optimal ratio of Ni^{2+} to protein was applied as 8. The L_n, L_w, PDI, and N values indicate number average length, weight average length, polydispersity index, and the number of counted 1D-assembled rods, respectively. The raw data in Supplementary Figure 32b are provided as a Source Data file.

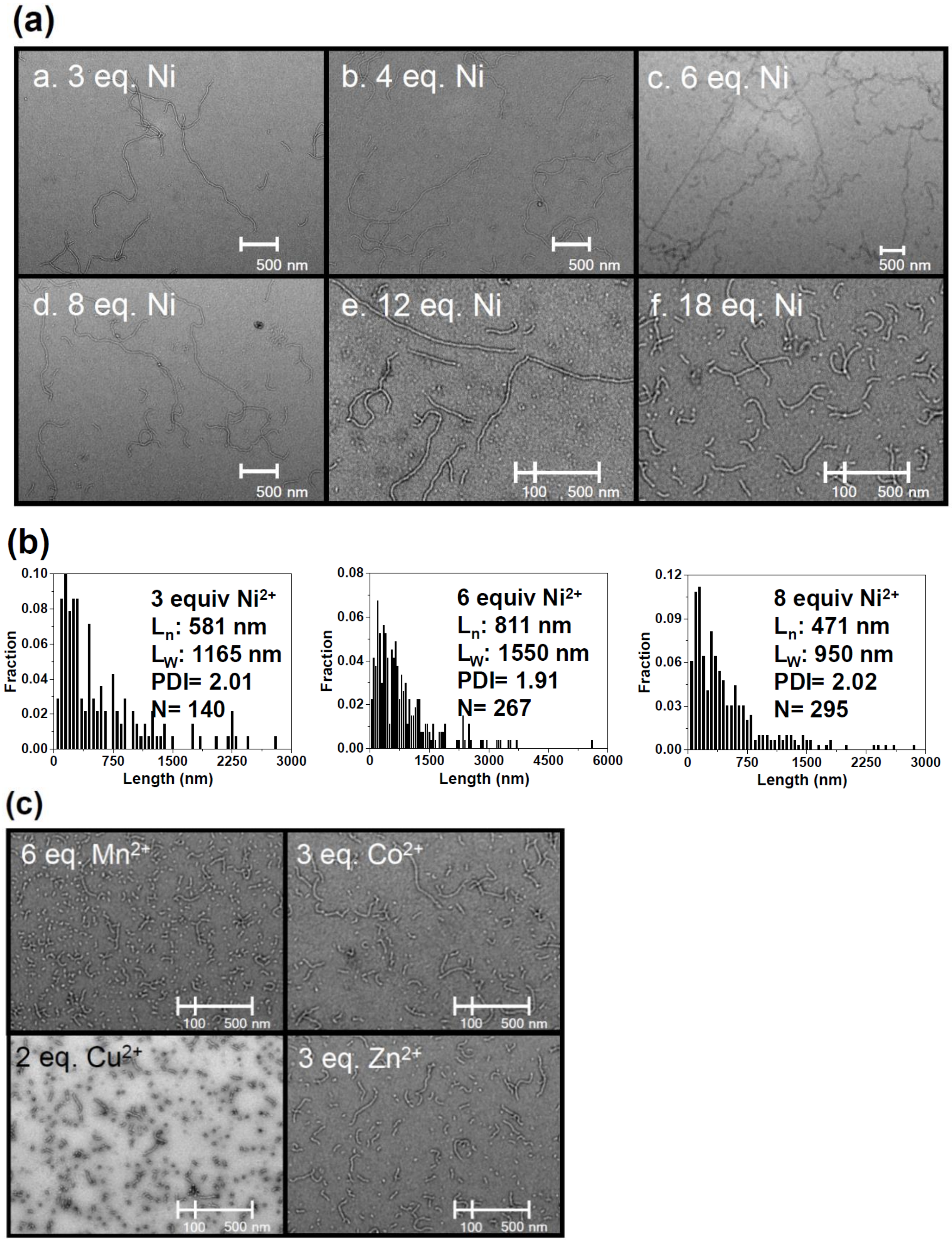

Supplementary Figure 33. Formation of 1D-rods with E13Z/K44E and Ni^{2+}. (a) TEM images. (b) Distributions of the 1D-assembled structures from E13Z/K44E (10 μM) with Ni^{2+} (3–18 equiv.) The L_n, L_w, PDI, and N values indicate number average length, weight average length, polydispersity index, and the number of counted 1D-assembled rods, respectively. (c) TEM images of E13Z/K44E reacted with other divalent metal ions. The raw data in Supplementary Figure 33b are provided as a Source Data file.

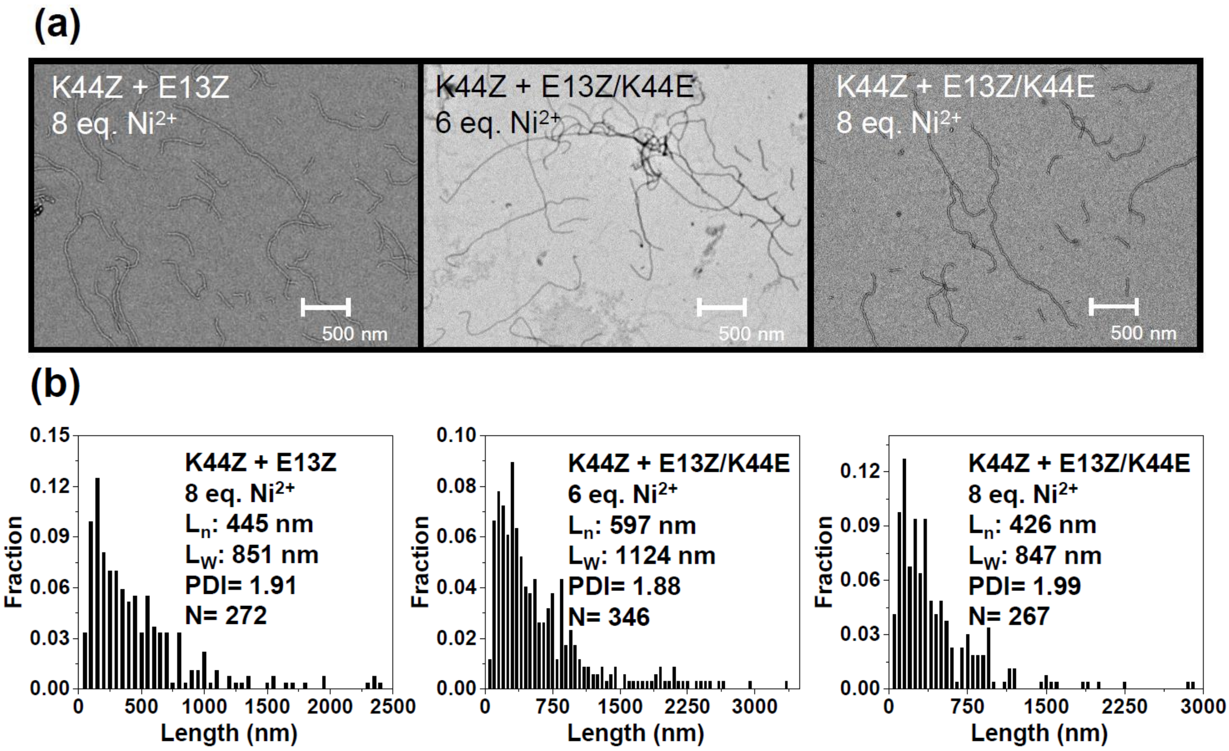

Supplementary Figure 34. Formation of 1D-asembled structures with two variants, K44Z and E13Z or E13Z/K44E. (a) TEM images. (b) Distributions from the data in (a). The L_n, L_w, PDI, and N values indicate number average length, weight average length, polydispersity index, and the number of counted 1D-assembled rods, respectively. The equal ratio of each protein (10 µM at final concentration) was pre-mixed, followed by the addition of. 6 or 8 equiv. Ni^{2+} at 37 ºC for 24 h. The raw data in Supplementary Figure 34b are provided as a Source Data file.

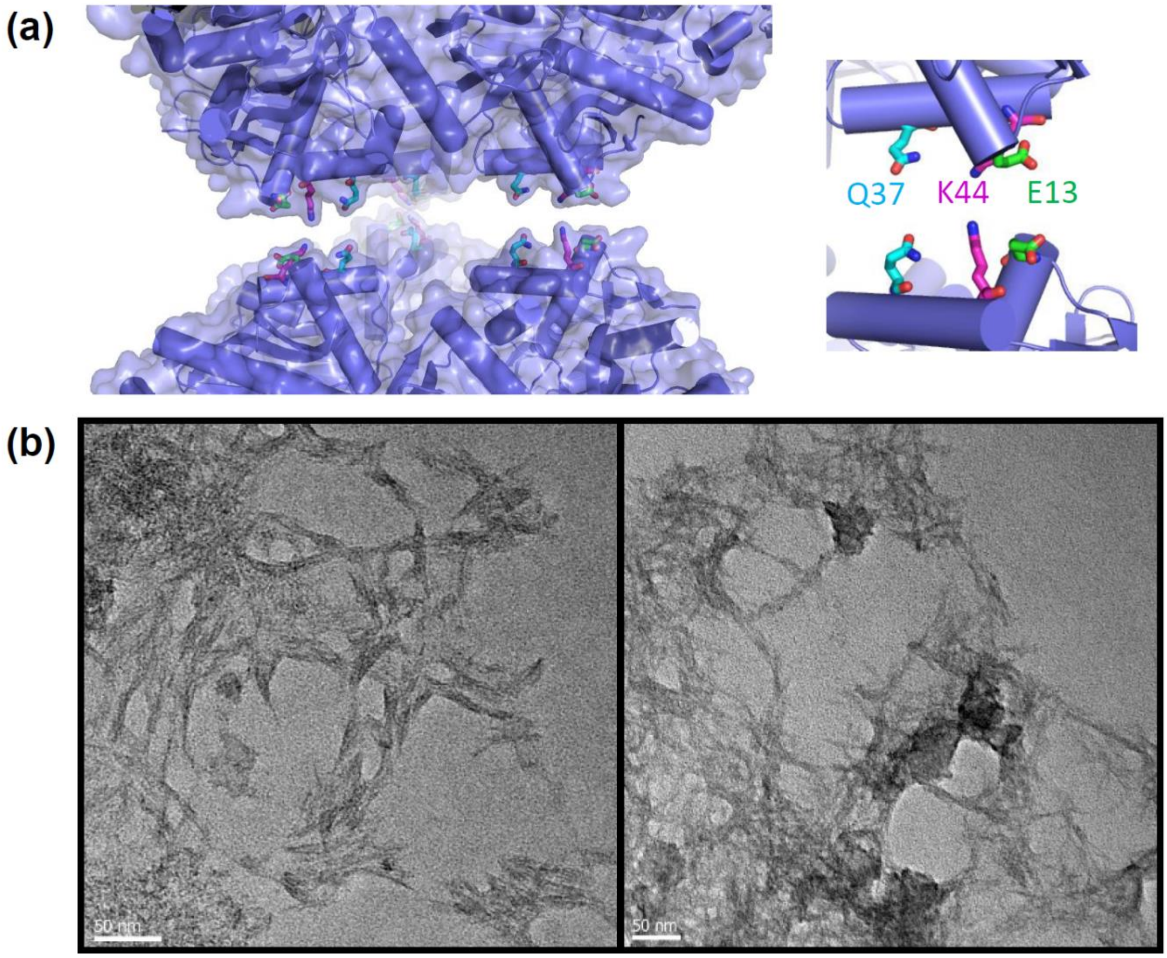

Supplementary Figure 35. Nanorod formation with Q37Z. (a) Position of Q37Z relative to K44 and E13 residues. (b) TEM images of Q37Z (50 μM) with 3 equiv of Ni^{2+} at 22 ºC for 24 h (Scale bar: 50 nm)

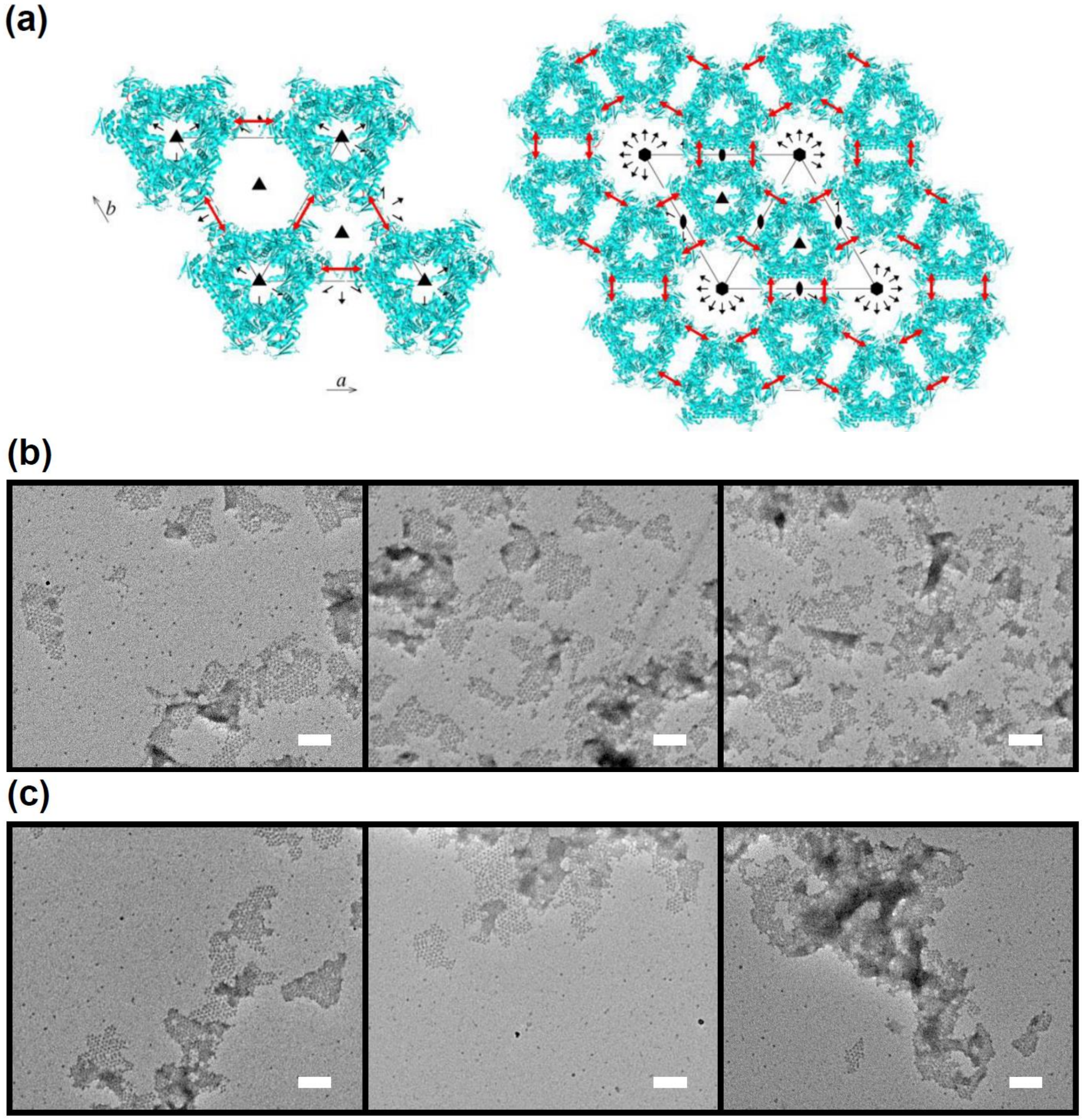

Supplementary Figure 36. Formation of 2D-planes with K337Z and Ni^{2+}. (a) Two potential layer symmetries assembled with the D_3 protein: p312 (left) and p622 (right). TEM images with (b) 5 μM (c) 10 μM of K337Z protein and 6 equiv Ni^{2+} at 37 °C for 24 h (Scale bar: 100 nm)

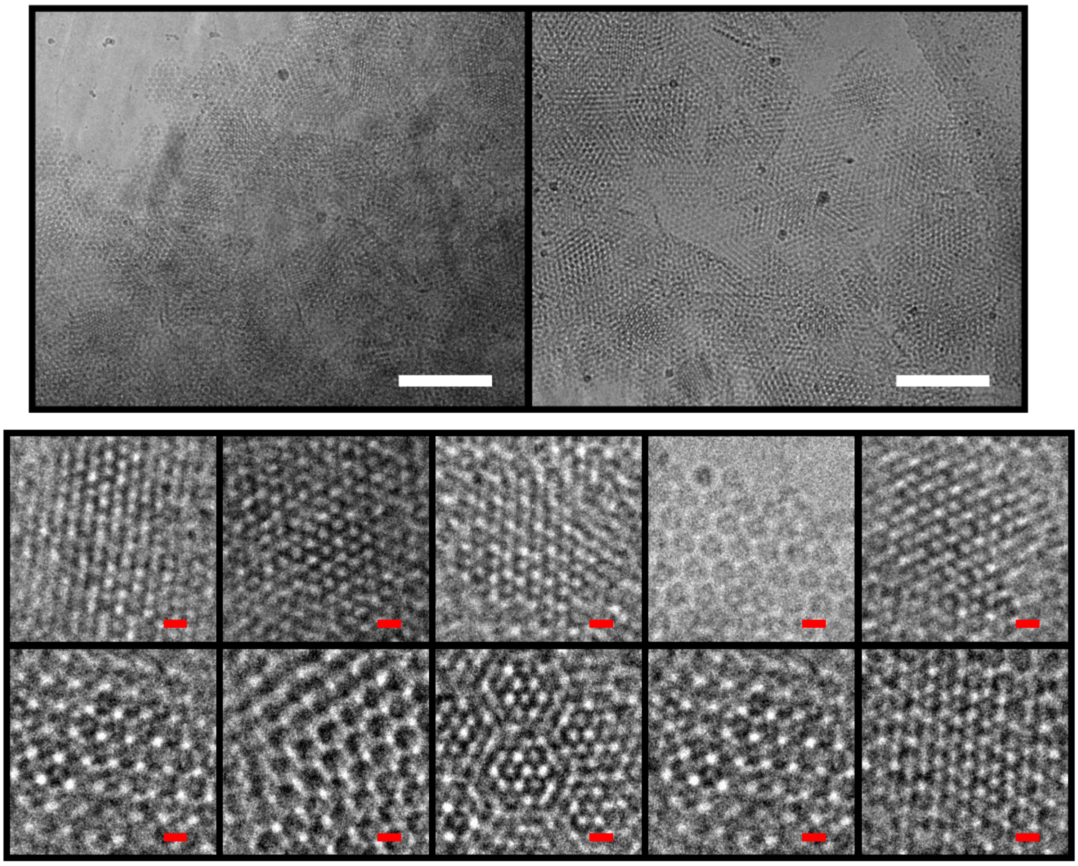

Supplementary Figure 37. Cryo-TEM images of 2D-planes from the reactions of K337Z with 6 equiv Ni^{2+} at 37 ^{o}C for 24 h (Scale bar: 200 nm for white, 10 nm for red)

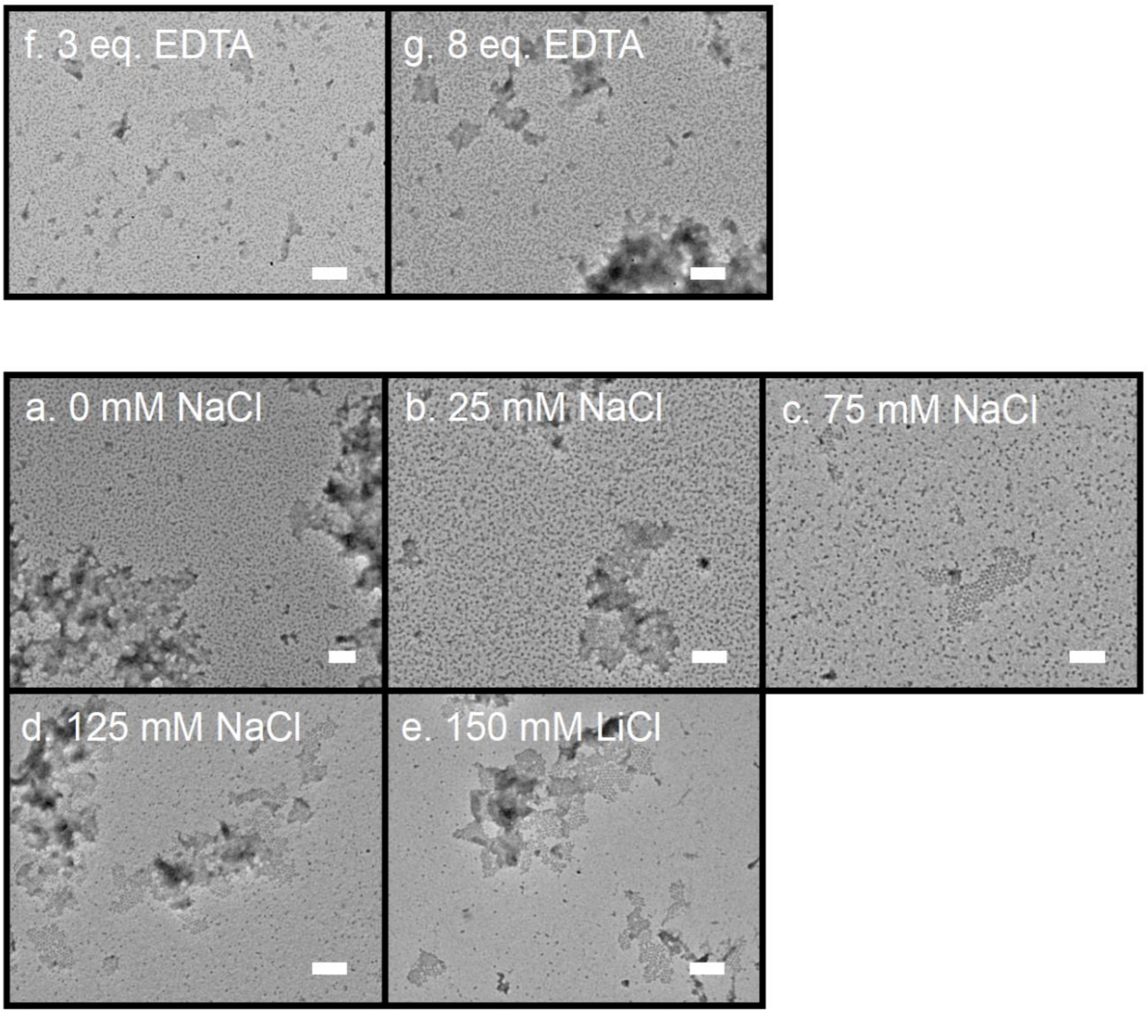

Supplementary Figure 38. TEM images of K337Z protein (10 μM) reacted with 6 equiv. of Ni^{2+} under various conditions. (a-d) [NaCl] = 0–125 mM NaCl (e) 150 mM LiCl (f-g) Destruction of pre-formed planes shown in Fig. S27-28 upon the addition of 3 or 8 equiv. of EDTA (Scale bar: 100 nm)

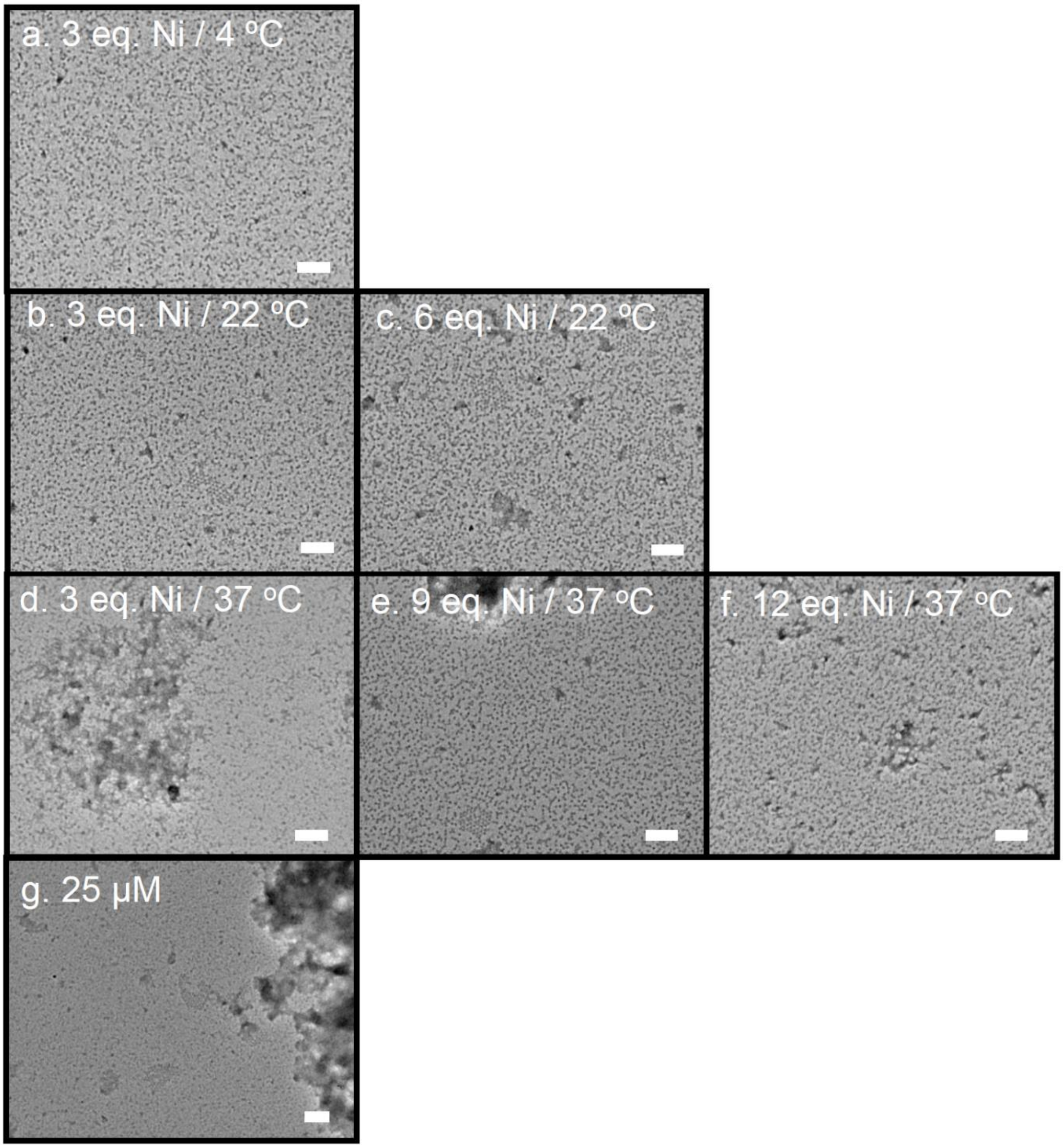

Supplementary Figure. 39 TEM images of 2D-planes under various conditions. [K337Z] = 5–10 µM reacted with (a) 3 equiv Ni^{2+} at 4 ºC (b–c) 3–6 equiv Ni^{2+} at 22 ºC (d–f) 3–12 equiv Ni^{2+} at 37 ºC (g) [K337Z] = 25 µM mixed with 6 equiv Ni^{2+} at 37 ºC (Scale bar: 100 nm)

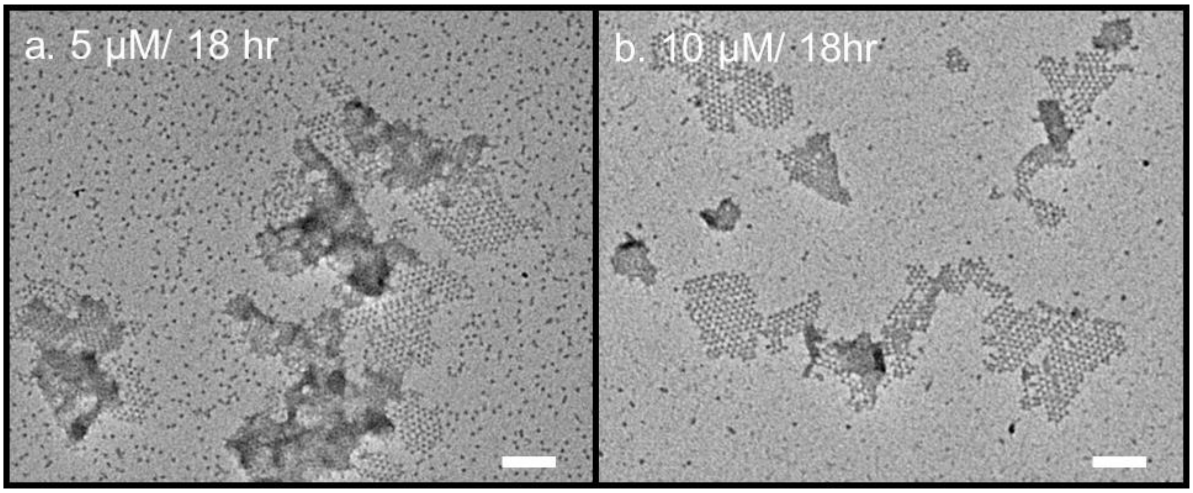

Supplementary Figure 40. TEM images of K337Z-derived planes formed in 18 h. (a) 5 μM (b) 10 μM of K337Z proteins. Relative to the data shown in the Supplementary Figures 27 (b) and (c), respectively, considerable amounts of unnreacted monomers were observed in (a). In contrast, no significant difference in the distribution of the monomers was observed in (b) (Scale bar = 100 nm)

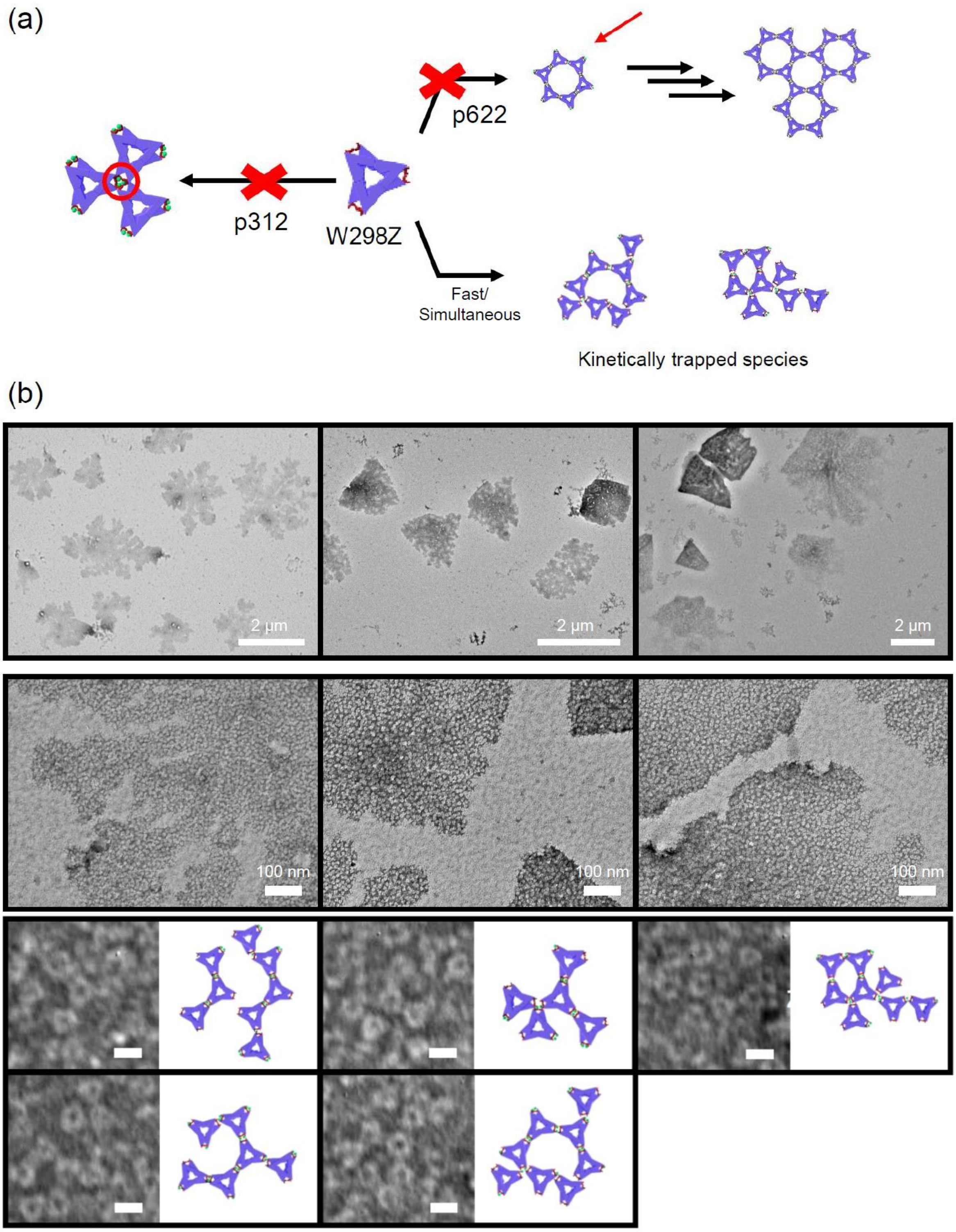

Supplementary Figure 41. Formation of noncrystaline 2D-materials with W298Z with Ni^{2+} (a) A scheme for 2D-spatial arrangements. Due to the relative location of W298Z, p312 is likely to be disfavored due to the resulting steric hindrance. p622 might be kinetically unfavored, due to the complexed nucleation step. (b) TEM images of W298Z protein (5-10 µM) with 10 eqiv. of Ni^{2+} at 22 °C.Only kinetically accessible, fractal-shaped 2D-structures were obtained. (Scale bar = 10 nm)

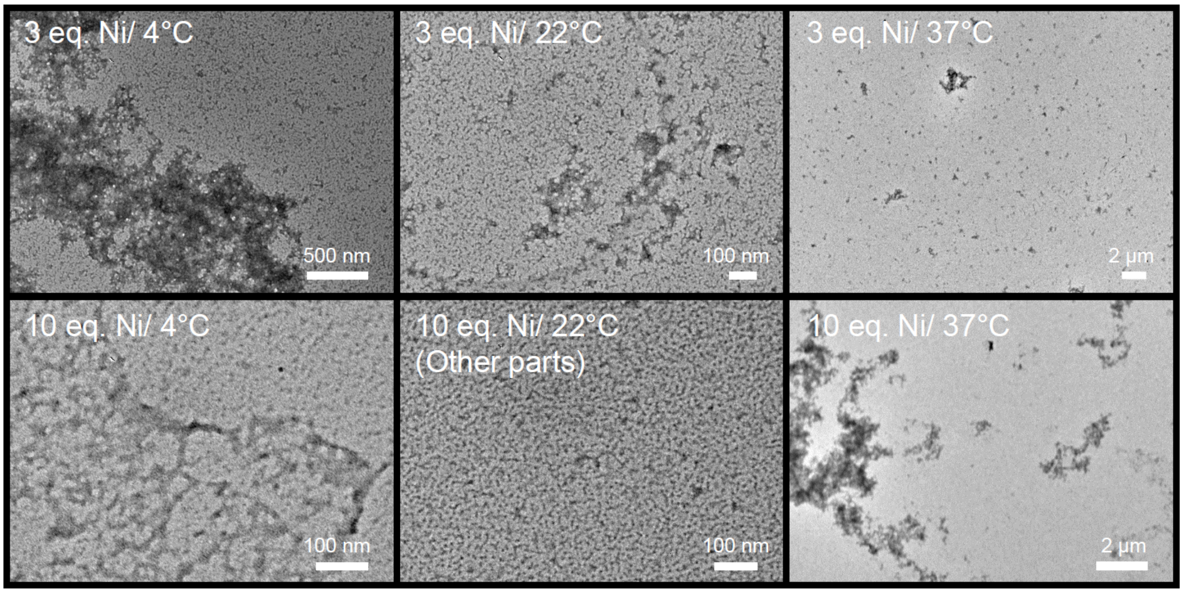

Supplementary Figure 42. TEM images of W298Z with Ni^{2+} under various conditions. Noncrysatlline, heterogeneous materials were observed in the listed conditions**.**

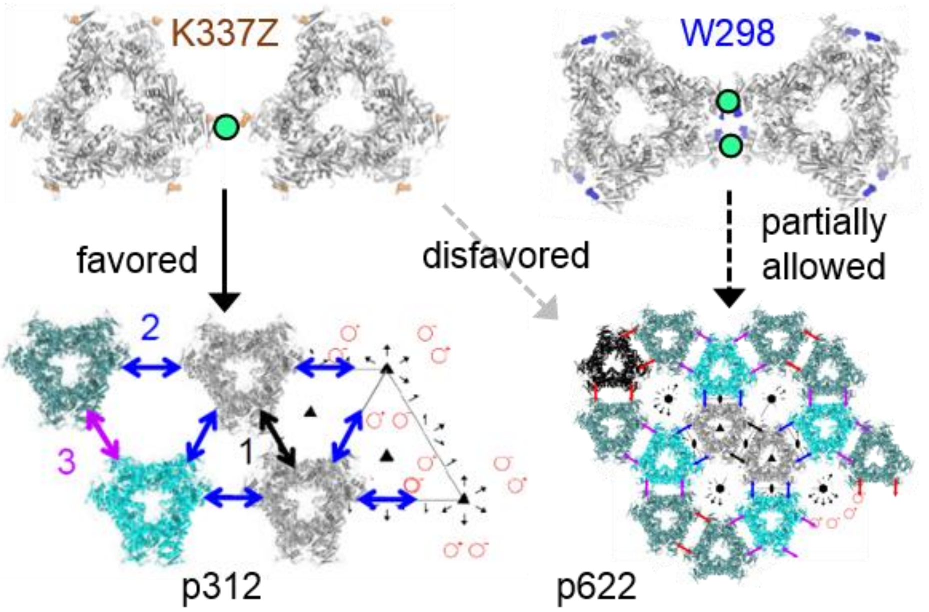

Supplementary Figure 43. Preference of K337Z and W298Z for p312 and p622 layer symmetries in 2D-assembly, respectively. The sequential steps in nucleation were numbered as, 1 (black arrows), 2 (blue arrows), 3 (magenta arrows), and 4 (red arrows), respectively.

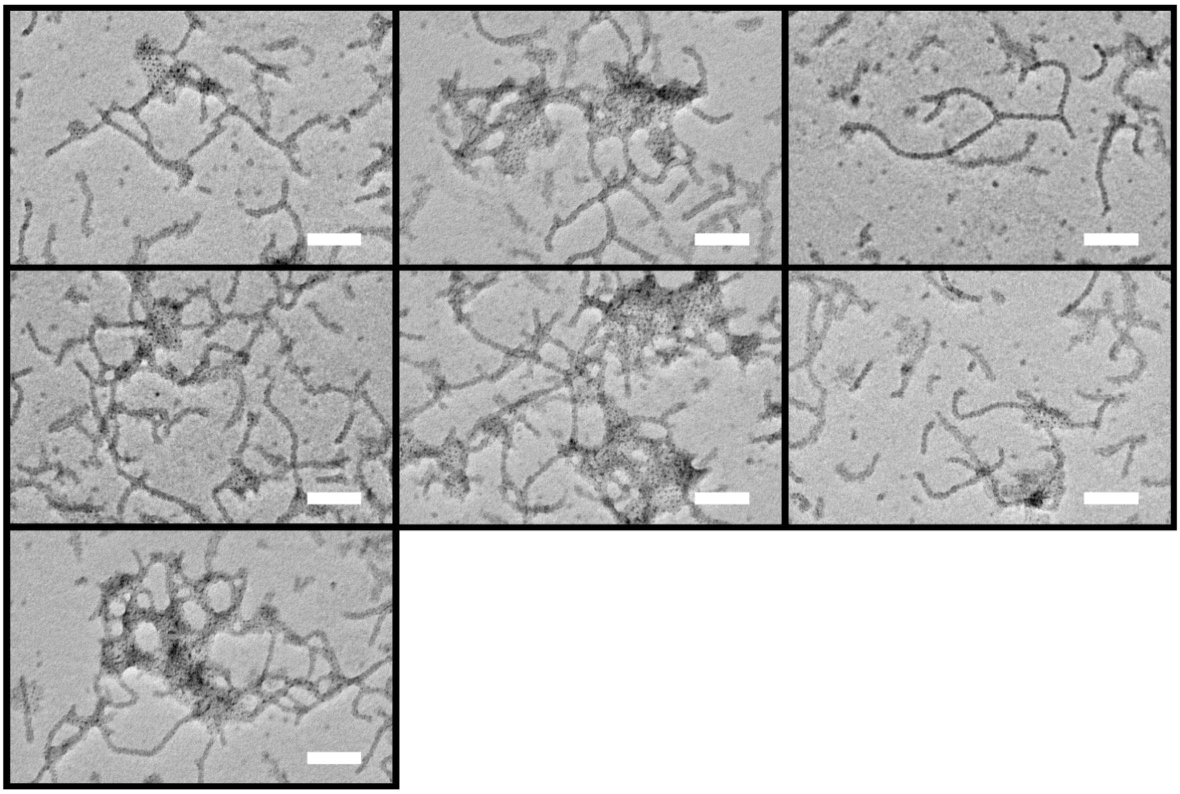

Supplementary Figure 44. TEM images of the hybrid structures with K44Z, K337Z, and Ni^{2+}. Relatively short nanorods were formed with K44Z (10 μM) with 3 equiv. of Ni^{2+} at 22 ºC for 4 h, followed by the addition of K337Z (2 μM) and 3 equiv. of Ni^{2+} 22 ºC for 20 h to yield desired structures.(Scale bar: 100 nm)

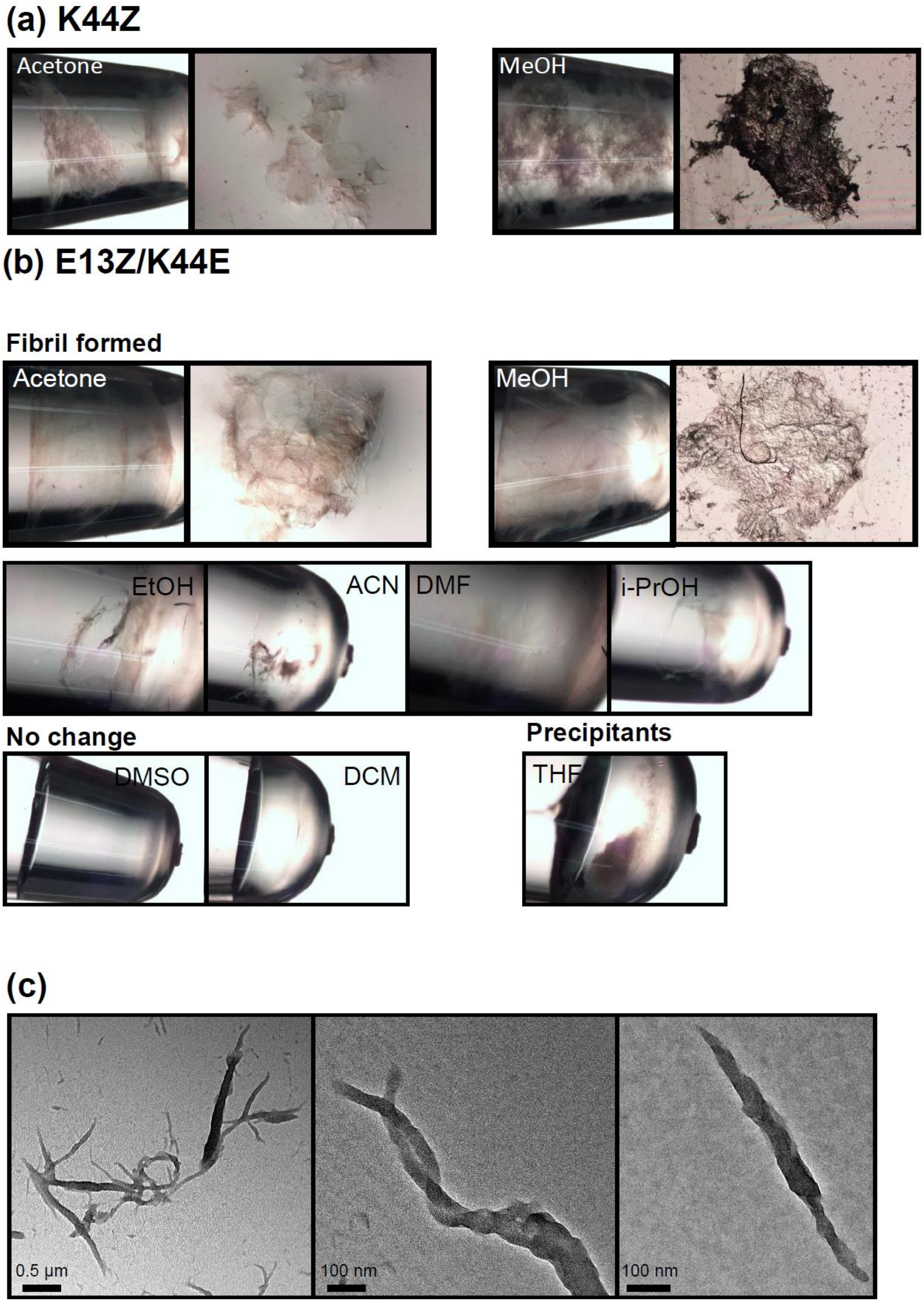

Supplementary Figure 45. Fibril formation with 1D-rods. Addition of organic solvents (4-fold volume to the buffer) to the pre-formed nanorods with (a) K44Z and (b) E13Z/K44E. K44Z-derived rods were aggregated upon the addition of acetone or methanol. E13Z/K44E-derived rods altered the overall structures depending on the applied solvents. (C) TEM images of fibrils with the K44Z-derived rods by the addition of uranyl acetate (0.2 wt. %)

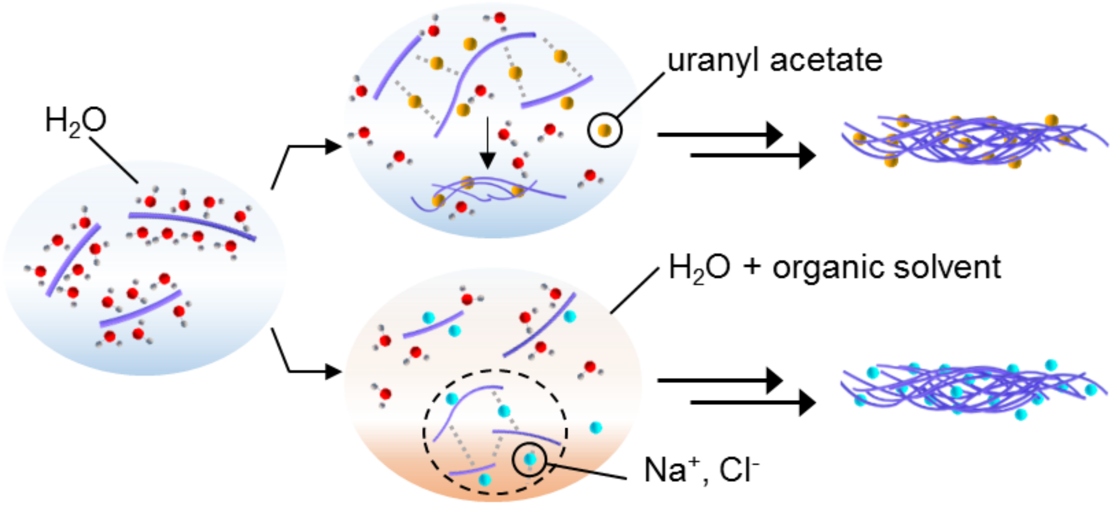

Supplementary Figure 46. A scheme for fibril formation with 1D-assembled products

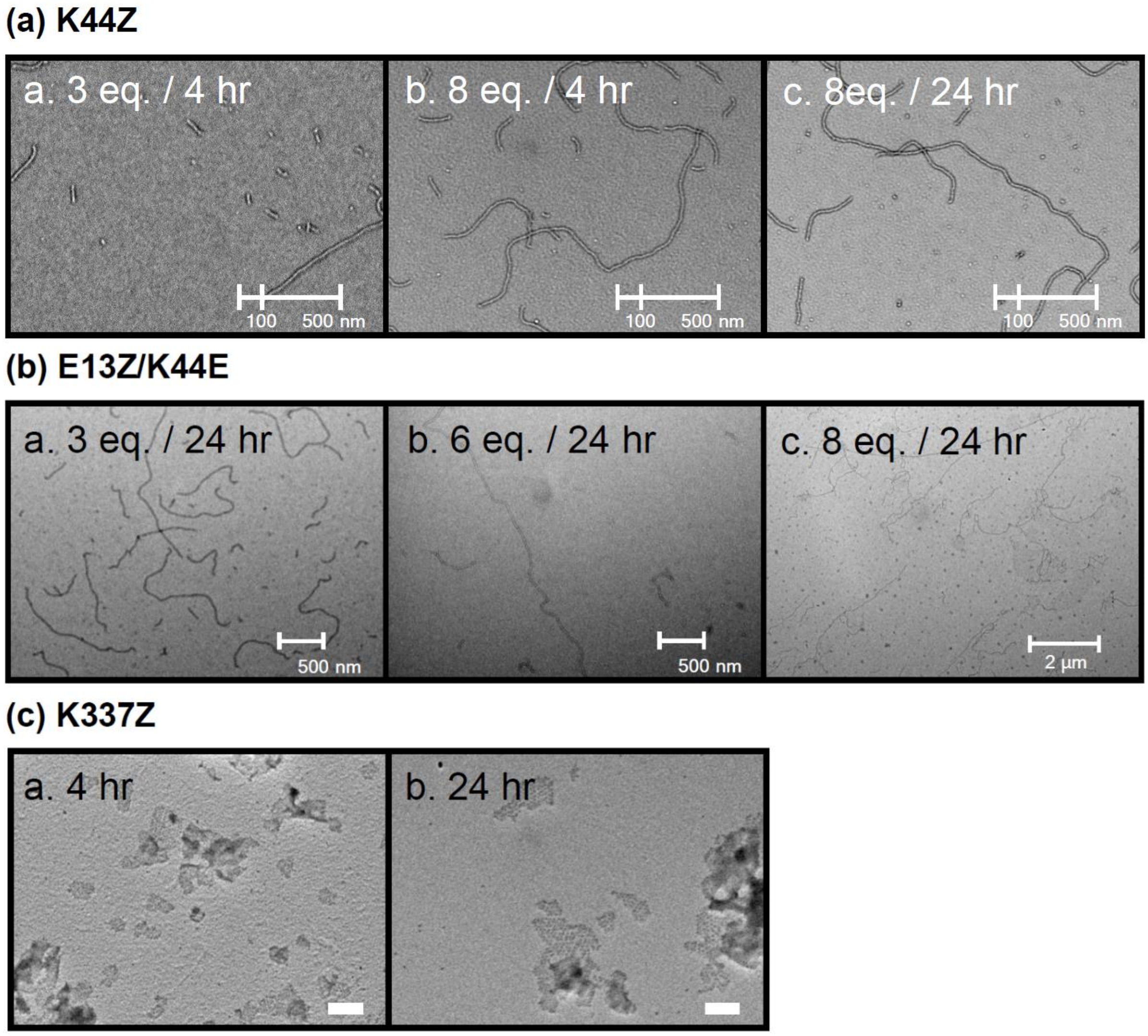

Supplementary Figure 47. TEM images of the 1D and 2D-assembled structures after heating at 55 °C. (a) K44Z (10 μM) rods were assembled with 3 or 8 equiv Ni^{2+} at 37 °C for 24 h and were incubated at 55 °C for 4–24 h. E13Z-derived rods were aggregated (not shown) whereas K44Z-derived rods became considerably shorter. (b) Pre-assembled rods with E13Z/K44E (10 μM) with various Ni^{2+} ratios to protein (3–8 equiv). With 8 equiv. of Ni^{2+}, the length of the rods heated at 55 ºC was longer than those at 37 ºC. (c). K337Z-derived 2D-planes with no considerable structural change upon heating for 4–24 h (scale bar: 100 nm)

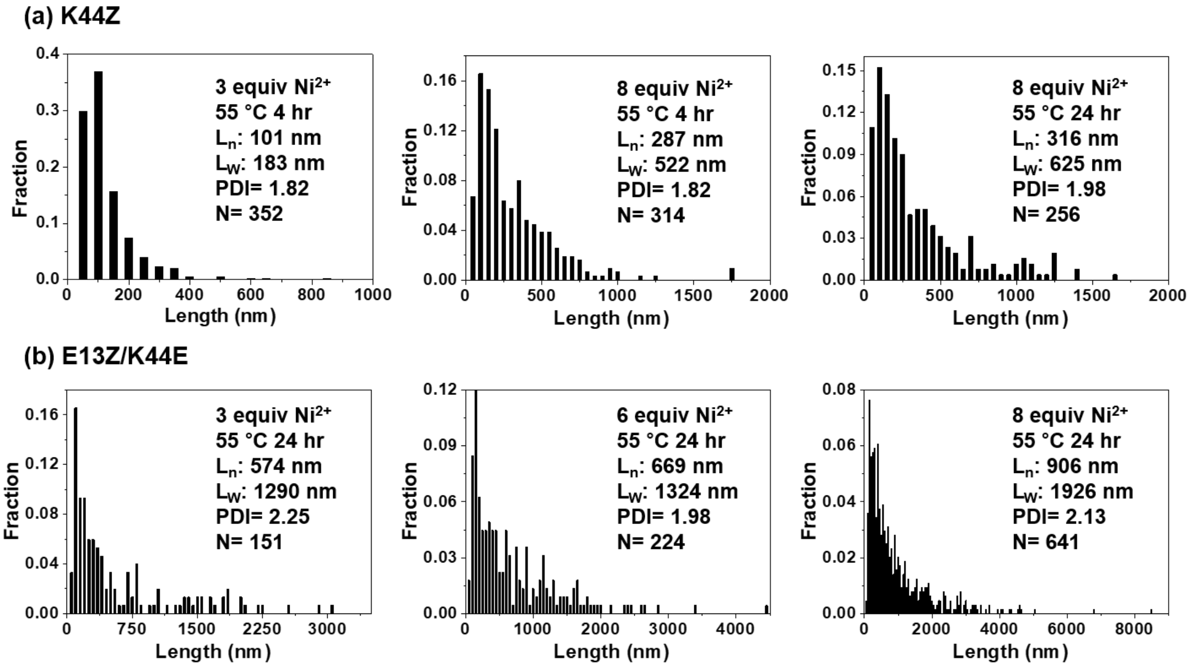

Supplementary Figure 48. Distributions of 1D-rods after heating at 55 °C shown in Supplemenatry Figure 47. The L_n, L_w, PDI, and N values indicate the number average length, weight average length, polydispersity index, and the number of counted 1D-assembled rods, respectively. (a) K44Z or (b) K44E/E13Z with 3–8 equiv. of Ni^{2+} were incubated for 24 h at 37 °C and further heated at 55 °C for 4–24 h. The raw data in Supplementary Figure 48 are provided as a Source Data file.

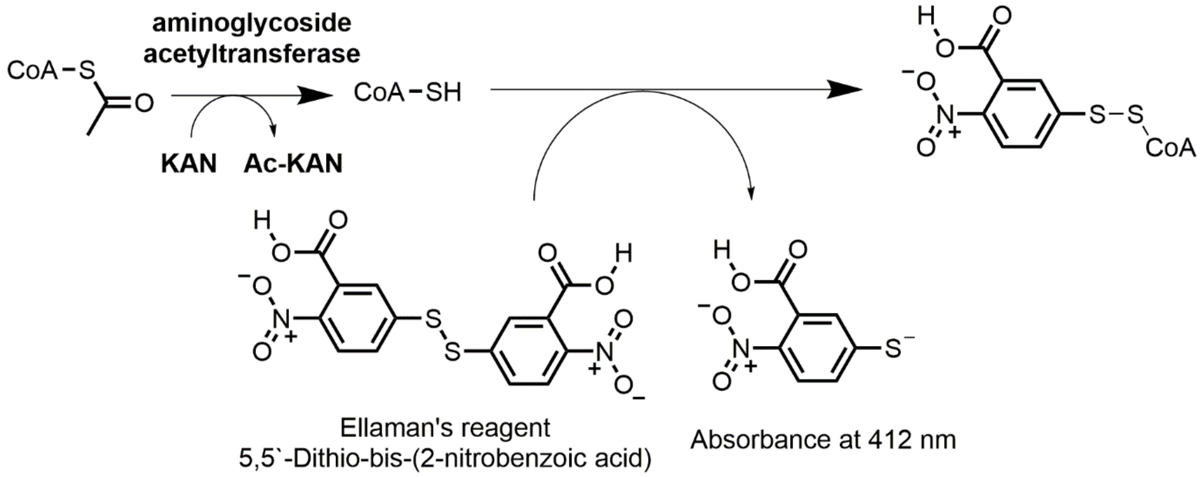

Supplementary Figure 49. The colorimetric assay to measure the activity of aminoglycoside acetyl-transferase

Supplementary Tables

Supplementary Table 1. Binding affinities of the listed divalent metal ions and bipyridine ligand at 25 °C, pH 7.0, ionic strength = 0[1,2]

M^{2+}	Log K_1	Log K_2	Log K_{OH-}
Mn^{2+}	4.06	3.78	3.4
Co^{2+}	5.65	5.60	4.3
Ni^{2+}	7.07	6.86	4.1
Cu^{2+}	8.15	5.50	6.3
Zn^{2+}	5.04	4.35	5.0

Supplementary Table 2. Sequence of the selected protein for the current work (PDB: 3N7Z)

Nucleotide sequences
**CATATG**AGCAATGCCATGAATGTGATTCGCCTGAAAGAAGATAAATTTCGCGAA<br>GCACTGCGTCTGAGCGAATATGCATTTCAGTATAAAGTGGATGAAGATCGTCTGC<br>AGCAGCAGATTACCAAAATGAAAGAAAGCCATGAAGTGTATGGCATCATGGAA<br>GGTGAAAATCTGGCAGCAAAACTGCATCTGATTCCGTTTCATATCTACATCGGCA<br>AAGAAAAATTCAAGATGGGTGGTGTTGCCGGTGTTGCAACCTATCCGGAATATC<br>GTCGTAGCGGTTATGTTAAAGAACTGCTGCAACATAGCCTGCAGACCATGAAAA<br>AAGATGGTTATACCGTTAGCATGCTGCATCCGTTTGCAGTTAGCTTTTATCGTAAA<br>TATGGTTGGGAACTGTGTGCCAATCTGCTGGTTTGTCACATGACCAAAAGCGAT<br>CTGGTTATGAAAAAACAGGTTAACGGCACCGTGAAACGCTTTAACAAAGAAAG<br>TCATCCGGAAGAGGTGGAAAAACTGTATGAAACCTTTGCAGAACTGTTTAGCG<br>GTATGCTGGTTCGTAATGAAAAATGGTGGCTGCAGGCAGTTTATGATGATCTGAC<br>CCTGGCAATCTATTATGATGAAAATCAGACCGCAGCAGGCTACATGCTGTATAAA<br>ATCGAGAACTATAAGATGACCGTGGAAGAATTTGTTCCGCTGCATAATGAAGCA<br>CGTAATGGTCTGTGGAACTTTATTTGTCAGCATGATAGCATGATCAAAGATCTGG<br>AAATGACCGTGAGCGAAAATGAACCGCTGCTGTATACCCTGCAAGAACCGCGT<br>GTTAAAACCGAAATTAAACCGTATTTTATGGGTCGCATTGTGGATGTTGAACAGT<br>TCCTGAAACAGTATGAACTGAATTGGAATAACGTGCAGCAAGAAGTGATTCTGC<br>ATATCACCGATAGCTTTGCACAGTGGAATAACATTACCGTTCGTATTGCCAACCA<br>TGAGATTACCATTATTGAAGAACCGATCGACAAAGGCATCAAACTGGATATTAAT<br>GCACTGAGCACCATCCTGTTTGGTTATCGTCGTCCGCTGGAACTGAATGAATTAG<br>AACTGATTAGTGGCAGCGAAGAAGAAATTCGCGCATTTGAAAGCGTTGTTCCGG<br>TTCGTAAACCGTTCATCTATGACTTTTTCTAA**CTCGAG**<br>* The cut-sites for restriction enzyme were highlighted in bold letters.
Protein sequences
SNAMNVIRLKEDKFREALRLSEYAFQYKVDEDRLQQQITKMKESHEVYGIMEGEN<br>LAAKLHLIPFHIYIGKEKFKMGGVAGVATYPEYRRSGYVKELLQHSLQTMKKDGY<br>TVSMLHPFAVSFYRKYGWELCANLLVCHMTKSDLVMKKQVNGTVKRFNKESHPE<br>EVEKLYETFAELFSGMLVRNEKWWLQAVYDDLTLAIYYDENQTAAGYMLYKIENY<br>KMTVEEFVPLHNEARNGLWNFICQHDSMIKDLEMTVSENEPLLYTLQEPRVKTEIK<br>PYFMGRIVDVEQFLKQYELNWNNVQQEVILHITDSFAQWNNITVRIANHEITIIEEPI<br>DKGIKLDINALSTILFGYRRPLELNELELISGSEEEIRAFESVVPVRKPFIYDFF<br>388 aa (Molecular weight: 45.7 *k*Da)

Supplementary Table 3. Primer sequences used for site-directed mutagenesis

Mutation	primer sequences (F = Forward, R = Reverse); 5'-sequence-3'
E13Z	F: GTGATTCGCCTGAAATAGGATAAATTTCGCG<br>R: CGCGAAATTTATCCTATTTCAGGCGAATCAC
Q37Z	F: GAAGATCGTCTGTAGCAGCAGATTACC<br>R: GGTAATCTGCTGCTACAGACGATCTTC
K44Z	F: CAGATTACCAAAATGTAGGAAAGCCATGAAGTG<br>R: CACTTCATGGCTTTCCTACATTTTGGTAATCTG
K44E	F: CAGATTACCAAAATGGAGGAAAGCCATGAAGTG<br>R: CACTTCATGGCTTTCCTCCATTTTGGTAATCTG
W298Z	F: CAGTATGAACTGAATTAGAATAACGTGCAGCAAG<br>R: CTTGCTGCACGTTATTCTAATTCAGTTCATACTG
N299Z*	F: GTATGAACTGAATTGGTAGAACGTGCAGCAAGAAG<br>R: CTTCTTGCTGCACGTTCTACCAATTCAGTTCATAC
N300Z*	F: GTATGAACTGAATTGGAATTAGGTGCAGCAAGAAGTG<br>R: CACTTCTTGCTGCACCTAATTCCAATTCAGTTCATAC
V301Z*	F: CTGAATTGGAATAACTAGCAGCAAGAAGTGATTCTG<br>R: CAGAATCACTTCTTGCTGCTAGTTATTCCAATTCAG
H326Z*	F: GTTCGTATTGCCAACTAGGAGATTACCATTATTG<br>R: CAATAATGGTAATCTCCTAGTTGGCAATACGAAC
E332Z*	F: GAGATTACCATTATTTAGGAACCGATCGACAAAG<br>R: CTTTGTCGATCGGTTCCTAAATAATGGTAATCTC
E333Z*	F: GATTACCATTATTGAATAGCCGATCGACAAAG<br>R: CTTTGTCGATCGGCTATTCAATAATGGTAATC
D336Z*	F: GAAGAACCGATCTAGAAAGGCATCAAACTG<br>R: CAGTTTGATGCCTTTCTAGATCGGTTCTTC
K337Z	F: GAAGAACCGATCGACTAGGGCATCAAACTGG<br>R: CCAGTTTGATGCCCTAGTCGATCGGTTCTTC

*The residues were poorly expressed with bpy-Ala in large scales and were not pursued.

Supplementary Notes

Supplementary Note 1. A series of reactions related to protein-assembly using bpy ligand. K_1, K_2, and K_{OH-} indicate the chemical equilibrium constants for Supplementary Equation (1)–(3), respectively.

$$[M(OH_2)_x] + bpy \leftrightarrow [M(bpy)] + xH_2O \cdots\cdots\cdots\cdots \text{ Supplementary Equation (1)}$$

$$[M(bpy)] + bpy \leftrightarrow [M(bpy)_2] \cdots\cdots\cdots\cdots \text{ Supplementary Equation (2)}$$

$$M + OH^- \leftrightarrow [M(OH)]^- \cdots\cdots\cdots\cdots \text{ Supplementary Equation (3)}$$

Supplementaty References

1 Irving, H. & Mellor, D. H. 1002. The stability of metal complexes of 1,10-phenanthroline and its analogues. Part I. 1,10-Phenanthroline and 2,2′-bipyridyl. *J. Chem. Soc.*, 5222-5237, doi:10.1039/JR9620005222 (1962).

2 Smith, R. M. & Martell, A. E. *Critical Stability Constants*. (Plenum Press, 1974).
